# Identifying gene expression profiles associated with neurogenesis and inflammation in the human subependymal zone from development through aging

**DOI:** 10.1038/s41598-021-03976-4

**Published:** 2022-01-07

**Authors:** Mainá Bitar, Christin Weissleder, Hayley F. North, Misaki S. Clearwater, Oressia Zalucki, Glenda M. Halliday, Maree J. Webster, Michael Piper, Cynthia Shannon Weickert, Guy Barry

**Affiliations:** 1 QIMR Berghofer Medical Research Institute, Herston, QLD 4006 Australia; 2 Schizophrenia Research Laboratory, Neuroscience Research Australia, Randwick, NSW 2031 Australia; 3 School of Psychiatry, Faculty of Medicine, University of New South Wales, Sydney, NSW 2052 Australia; 4 School of Biomedical Sciences, The University of Queensland, Brisbane, 4072 Australia; 5 School of Medical Sciences, Faculty of Medicine, University of New South Wales, Sydney, NSW Australia; 6 Neuroscience Research Australia, Sydney, NSW Australia; 7 Laboratory of Brain Research, Stanley Medical Research Institute, Bethesda, MD 20815 USA; 8 Queensland Brain Institute, The University of Queensland, Brisbane, 4072 Australia; 9 Department of Neuroscience and Physiology, Upstate Medical University, Syracuse, NY 13210 USA

**Keywords:** Adult neurogenesis, Neural stem cells

## Abstract

The generation of new neurons within the mammalian forebrain continues throughout life within two main neurogenic niches, the subgranular zone (SGZ) of the hippocampal dentate gyrus, and the subependymal zone (SEZ) lining the lateral ventricles. Though the SEZ is the largest neurogenic niche in the adult human forebrain, our understanding of the mechanisms regulating neurogenesis from development through aging within this region remains limited. This is especially pertinent given that neurogenesis declines dramatically over the postnatal lifespan. Here, we performed transcriptomic profiling on the SEZ from human post-mortem tissue from eight different life-stages ranging from neonates (average age ~ 2 months old) to aged adults (average age ~ 86 years old). We identified transcripts with concomitant profiles across these decades of life and focused on three of the most distinct profiles, namely (1) genes whose expression declined sharply after birth, (2) genes whose expression increased steadily with age, and (3) genes whose expression increased sharply in old age in the SEZ. Critically, these profiles identified neuroinflammation as becoming more prevalent with advancing age within the SEZ and occurring with time courses, one gradual (starting in mid-life) and one sharper (starting in old age).

## Introduction

Throughout life, the adult mammalian brain retains the ability to produce neurons, a process that occurs predominantly within two neurogenic niches within the forebrain; the subgranular zone of the hippocampal dentate gyrus, and the subependymal zone (SEZ) lining the lateral walls of the lateral ventricles (also known as the ventricular–subventricular zone or V-SVZ)^1,2^. Studies in rodents have provided extensive insights into the role of adult neurogenesis, linking this process to learning, memory, pattern separation and spatial navigation (hippocampal neurogenesis)^3^ and innate behaviours including maternal care and mating as well as associative learning (SEZ-derived neurogenesis)^4,5^. Ongoing neurogenesis has also been suggested to occur within the adult human brain^2,3^. Controversy still exists as to the extent of human adult neurogenesis in the hippocampal dentate gyrus. Whereas a number of studies have reported that neurogenesis is evident within the adult human dentate gyrus^6,7^, a recent study found no evidence of newborn neurons within the subgranular zone in humans post-adolescence^8^. Interestingly, this latter study did reveal the presence of immature neurons within the SEZ of adult humans^8^. Indeed, there is extensive evidence for the existence of ongoing neurogenesis within the postnatal human SEZ^9–14^. The question then arises as to the role of SEZ-derived neurogenesis in the mature brain. In rodents, neuroblasts generated from neural stem and progenitor cells within the SEZ migrate via the rostral migratory stream to the olfactory bulb, where they eventually differentiate into local interneurons^1,2^. The SEZ has also been recently shown to generate newborn medium spiny interneurons for the nucleus accumbens, a process that is also regulated by chronic pain^15^. In humans, on the other hand, the role of SEZ-derived adult neurogenesis is not clear. Migrating neuroblasts have been found both within the rostral migratory stream^9^ and throughout the frontal lobe^16,17^, in the first years of life. Although levels of neurogenesis and neurogenic markers drop dramatically within the human SEZ during early childhood^11,13,14,17^, numerous studies have reported ongoing neurogenesis within this neurogenic niche throughout adulthood and advanced age^8,12,18^, with immature neurons appearing to target the striatum rather than the olfactory bulb^18^.

Much of our understanding of neurogenesis within the adult SEZ has come from studies conducted in rodents. Within the rodent SEZ, an intricate niche environment including ependymal cells, blood vessels, astrocytes and microglia, collectively regulate neural stem cell activity^1,2^. This niche is also influenced by input from other brain regions, such as the hypothalamus^19^. Recent single-cell RNA sequencing studies have highlighted the diverse range of cells within the adult rodent SEZ^20–22^. The majority of neural stem cells within the mature SEZ are quiescent, a process regulated, in part, by Notch2^23^. Once activated, these neural stem cells divide symmetrically, to either self-renew or to ultimately generate neurons^24^. Approximately 80% of these symmetric divisions are consumptive, generating transit-amplifying cells, which divide rapidly before generating immature neurons or glia. As such, there is a marked decrease in neural stem cell number, and neurogenesis, over the course of development and aging in the rodent SEZ. Indeed, the aging SEZ undergoes a range of alterations, including a reduction in the number of ependymal cells and thinning of the niche^1^. Using immunocytochemistry and bulk RNA sequencing, a recent study confirmed a sharp decrease in neurogenesis in the mouse brain at 18 months in comparison to 2 months^25^. Moreover, this study revealed elevated inflammation as a hallmark feature of the aged SEZ niche in mice^25^, from which we hypothesized that transcripts associated with neuroinflammation would be increased in the aged SEZ neurogenic niche in humans.

Increased levels of inflammation interfere with neurogenesis within the adult mouse hippocampus, with cranial irradiation resulting in a marked increase in activated microglia, concomitant with reduced hippocampal neurogenesis^26^. Moreover, blocking the inflammatory response, both in models of cranial irradiation^27^ and in models of Alzheimer’s disease^28^, can restore hippocampal neurogenesis. Within the SEZ, inflammatory pathways have also been shown to suppress adult neurogenesis and to impair olfactory behavior^29,30^. Additionally, in rodents, microglia in this neurogenic niche have been shown to become progressively more active over the course of aging^31^, suggestive of a direct contribution, or indirect response, to the decline in neurogenesis over the course of aging.

One question arising from the results found in the developing and aging mouse brain is: How reflective are these changes that occur over 18 months to the cellular and molecular changes that occur over decades within the human SEZ? We have previously used post-mortem human samples to demonstrate that neurogenesis continues throughout the lifespan within the human SEZ^11–14^, and that neurogenesis declines during postnatal development^14^, accompanied by decreased mRNA expression of trophic factors and cognate receptors including *TRKB* and *IGF1*^13,32^*.* During aging, we see a further reduction in *IGF1* as well as increased expression of truncated *TRKB* and of the microglial marker *IBA1*^12,13,32^. In order to place changes in neurogenesis markers in a larger context and identify key changes in the microenvironment that co-occurred with shifts in neurogenic markers, we employed a global analysis of transcriptional architecture over the human life span. To address this, we used our unique cohort of SEZ samples from 3 days to 103 years of age and performed bulk RNA sequencing after targeted dissection of the SEZ. To analyze these data, we employed an integrated computational pipeline, which allowed us to assess gene expression and cluster genes with similar trajectories over the course of life. This approach identified a range of different expression profiles, three of which we focus on: (1) genes whose expression declined sharply after birth, (2) genes whose expression increased steadily over the course of aging, and (3) genes whose expression increased sharply in old age. Collectively, these results provide a novel insight into the transcriptional profiles within the human SEZ over the lifespan. Furthermore, this is a rich resource for the research community to build upon in order to understand the mechanisms contributing to age-related changes within this neurogenic niche.

## Results

### Relationships between demographic variables in the RNA sequencing and qPCR validation cohorts

**Figure 1:**
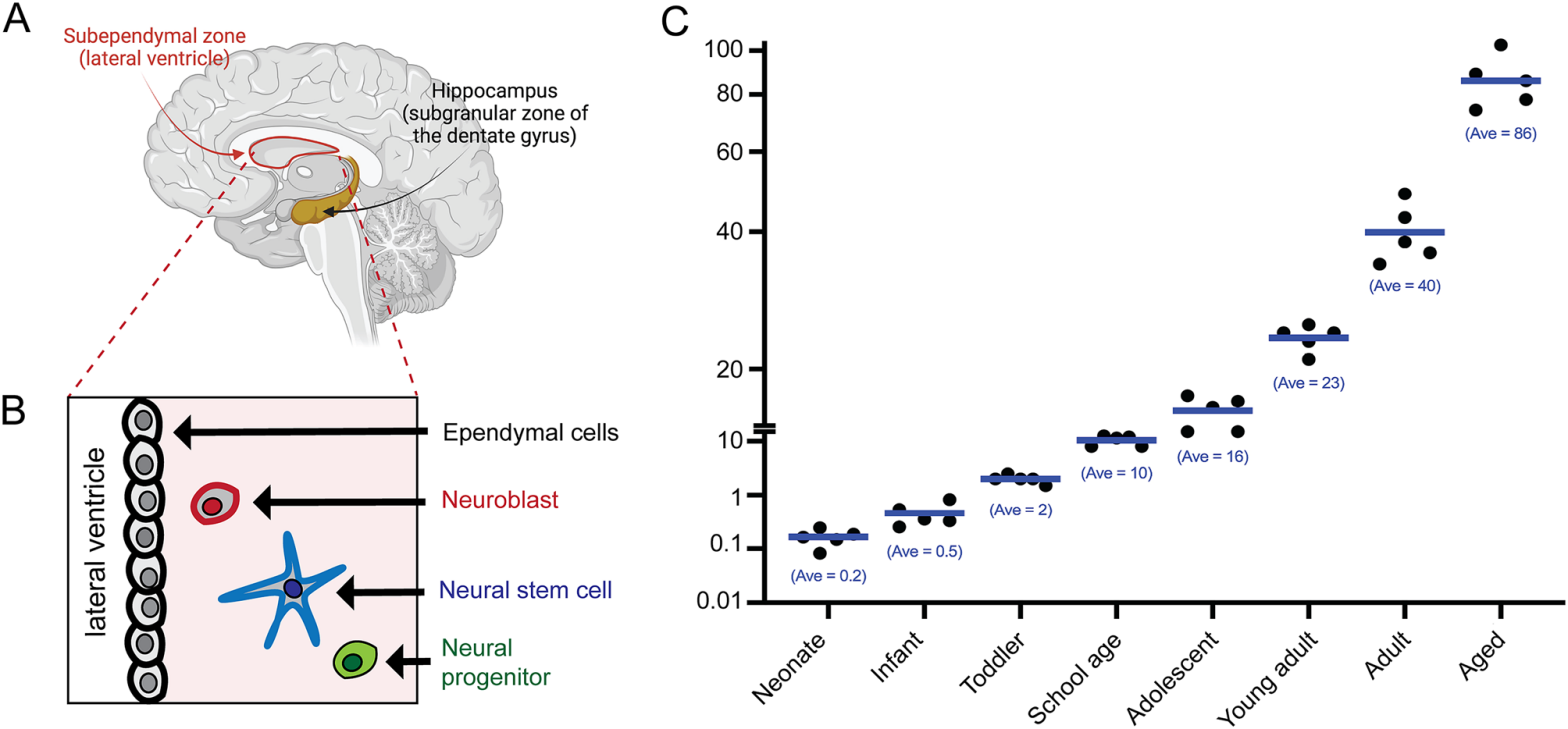
Cohort used to generate a transcriptional atlas of gene expression within the human SEZ grouped by age. (**A**) Schematic of a mid-sagittal view of a human cerebral cortex, depicting the two main adult neurogenic niches, the subependymal zone (SEZ), and the subgranular zone of the hippocampal dentate gyrus. (**B**) Some of the principal cell types found within the SEZ. (**C**) Graph showing the age of the samples from our RNA sequencing cohort. Samples were grouped into the following groups: neonate, infant, toddler, school age, adolescent, young adult, adult and aged adult. The average age of the samples in years is shown below the data points for each group. We sequenced dissected SEZ tissue from five samples in each age group. Panel (**A**) was generated using BioRender.

**Figure 2:**
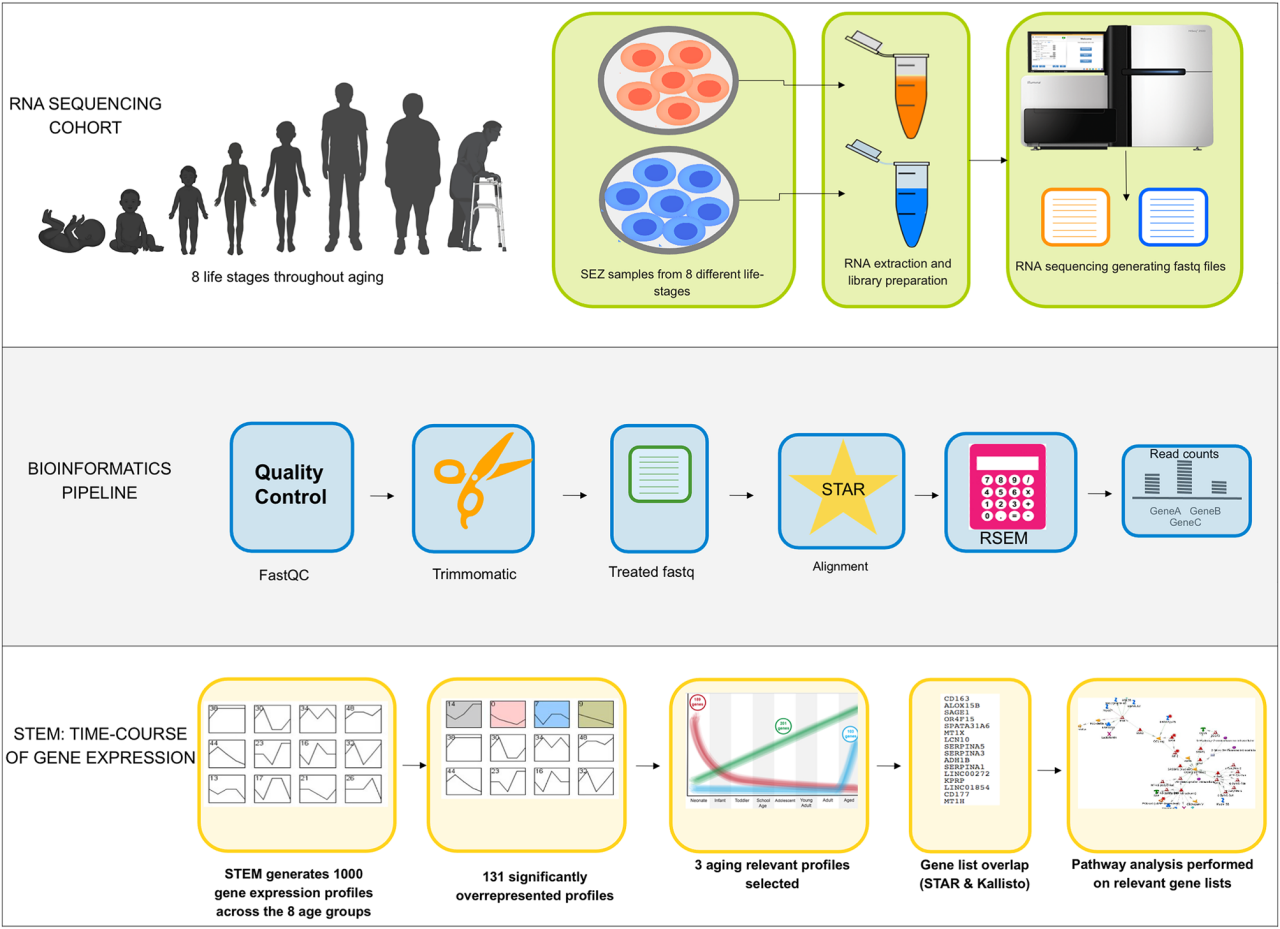
Overview of the steps employed in the sequencing and bioinformatic pipeline used in this study. The silhouette image of the age groups was generated using BioRender.

To define how gene expression changes within the human SEZ over the course of development and aging, we isolated RNA from the SEZ that was microdissected from along the lateral wall of the lateral ventricle. The cohort was assembled from brain samples derived from The University of Maryland Brain and Tissue Bank (Baltimore, USA), the New South Wales Brain Tissue Resource Centre (Sydney, Australia) and the Sydney Brain Bank (Sydney, Australia). This RNA sequencing cohort encompassed eight different life stages: neonate, infant, toddler, school age, adolescent, young adult, adult and aged adult (Fig. 1). We used a scalpel to dissect the SEZ from fresh-frozen tissue from the anterior third of the caudate nucleus (see “Methods” section). RNA was extracted from the samples, which were then processed for bulk RNA sequencing, followed by bioinformatic processing and interrogation (Fig. 2). We also used samples from a larger cohort to perform qPCR-based validation (see “Methods” section).

The RNA sequencing cohort (n = 5 per group, 8 groups, total n = 40) is described in Supplementary Table 1, including age at death, brain pH, post-mortem interval (PMI; in hours), RNA integrity number (RIN), sex and race. Interestingly, in this cohort, the aged group had a lower brain pH than all other groups except adulthood (ANOVA, F(7,39) = 2.52, *p* = 0.35, post hocs *p* < 0.03). RIN, PMI and sex did not significantly differ between groups (all *p* > 0.09, data not shown). Age at death negatively correlated with brain pH (r = − 0.53, *p* < 0.01), suggestive of acidification within the aged brain as reported previously^33^, but did not correlate with RIN or PMI (all *p* > 0.45).

Details of the qPCR validation cohort (n = 10 per group, 7 groups, total n = 70) are shown in Supplementary Table 2. In this cohort, brain pH and PMI were significantly different across age groups (all *p* ≤ 0.04). Brain pH was significantly higher during infancy, childhood, adolescence, young adulthood and adulthood compared to aging (all *p* ≤ 0.04). PMI was significantly longer in the neonatal group compared to infancy, childhood, adolescence, young adulthood and adulthood (all *p* ≤ 0.007). PMI was significantly longer in the aging group compared to childhood, adolescence, young adulthood and adulthood (all *p* ≤ 0.05). RIN and sex did not significantly differ between age groups (all *p* > 0.05, data not shown). Age at death negatively correlated with brain pH (r = − 0.31, *p* = 0.009). RIN positively correlated with brain pH (r = 0.54, *p* < 0.001). Age at death did not correlate with RIN or PMI (all *p* > 0.05).

### Relationships between demographic variables and target gene expression in the qPCR validation cohort

Expression of housekeeping genes *ACTB*, *GAPDH* and *UBC* and their geometric mean positively correlated with brain pH and RIN, demonstrating that the geometric mean is an appropriate normalizing factor (Supplementary Table 3, all r/ρ ≥ 0.27, *p* < 0.03). *DLX1*, *TLR2* and *IGF1* mRNAs positively correlated with PMI, while *CD163* mRNA negatively correlated with brain pH (Supplementary Table 3). No other significant relationships were observed between demographic variables and target gene expression.

### Age-related changes in gene expression profiles within the SEZ

Briefly, sequenced samples were computationally processed to remove adapter sequences and lower quality bases from reads and further filtered by minimum read length. Trimmed and filtered read pairs were subsequently aligned to the human transcriptome using STAR and transcript counts levels were estimated using RSEM (Fig. 2; see “Methods” section). Isoform abundance was collapsed into gene expression by direct addition of transcript counts. A gene expression matrix was created, integrating all replicates and life-stages, and used as input to STEM, a method for clustering genes into temporal expression profiles. Significant profiles (*p* < 0.05, with Bonferroni correction) are reported by STEM in a colour-coded graphical format, depicting expression level across life-stages of genes clustered by temporal similarity. Analysis of our novel and unique dataset with our bioinformatic pipeline discovered more than 130 significant time-course expression profiles. Three of these profiles were chosen as the focus of this study for multiple reasons, namely (1) the high number of genes they encompassed, (2) the common functions of these genes and (3) the distinct temporal expression pattern of these profiles, all of which point to their potential relevance to the aging process.

### Genes that decreased rapidly during early childhood

**Figure 3:**
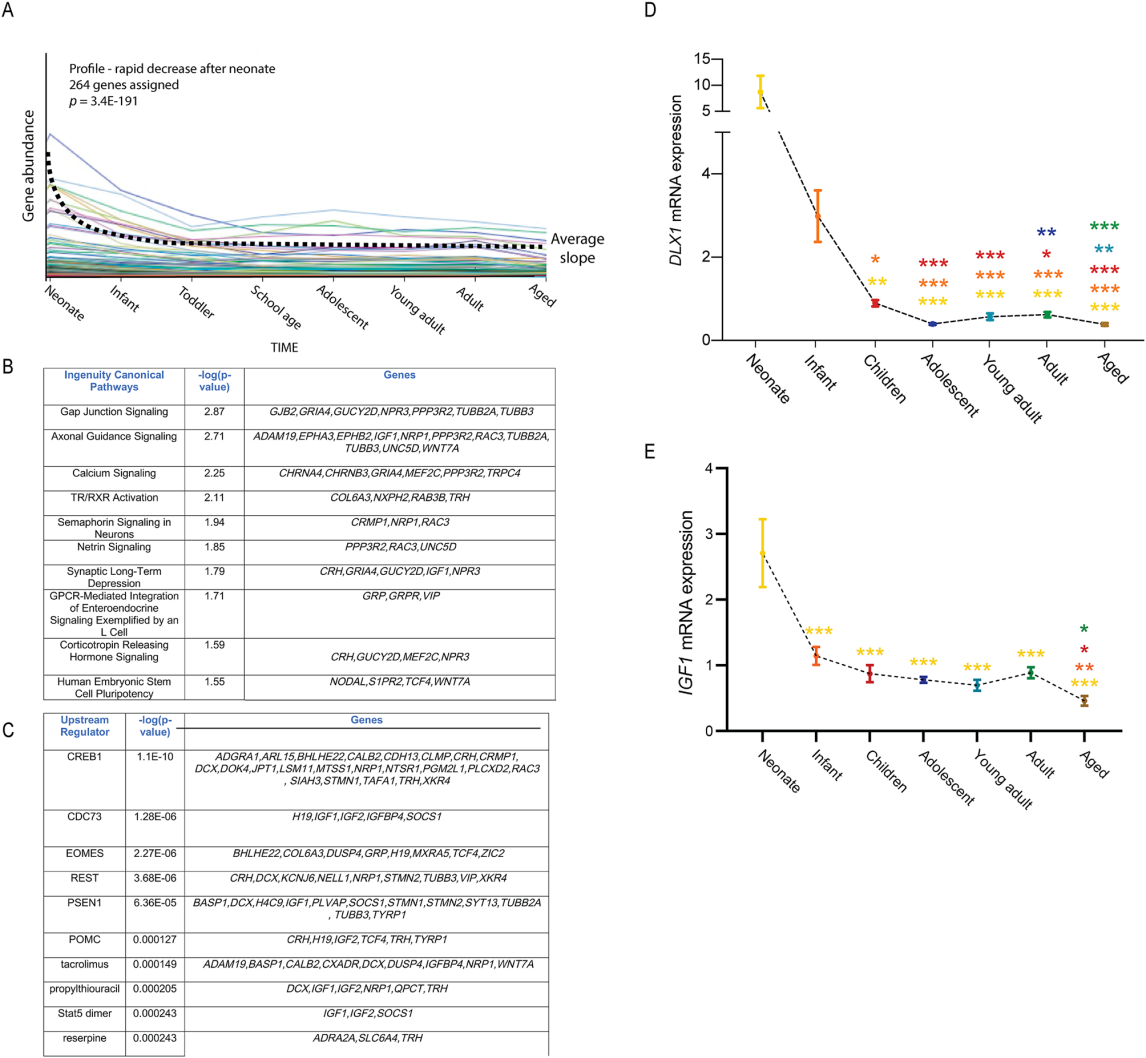
Transcripts whose expression fell rapidly after birth. (**A**) The first major aging-relevant profile that we identified was one in which gene expression dropped rapidly after birth. There were 264 genes associated with this profile. (**B**) Pathway analysis revealed many neuron-associated pathways linked to this profile. (**C**) Potential upstream regulators of the genes associated with this profile as determined through Ingenuity Pathway Analysis. (**D**,**E**) qPCR confirmed significantly reduced levels of *DLX1* (**D**) and *IGF1* (**E**) mRNA within the SEZ of older samples. Colours for significance (***) correspond to the colour of the age group used in the comparison. *p < 0.05, **p < 0.01, ***p < 0.001; Quade’s rank analysis of covariance or analysis of covariance, post hoc tests using LSD.

The first of these profiles followed a trajectory whereby gene expression was high in neonates, but dropped rapidly during infancy, tended to reach plateau levels by toddlers and subsequently stayed low throughout the rest of postnatal and adult life (Fig. 3A). There were 264 genes under the umbrella of this profile (Supplementary Table 4). Our findings are consistent with literature data which report that, both in rodents and humans, neurogenesis within the SEZ neurogenic niche decreases with aging^9,17,24^. Indeed, many genes associated with stem cell maintenance and neurogenesis were captured in this profile. Further analysis of the gene network represented by this profile, combining both physical and functional interactions, identified several developmental processes (e.g. anatomical structure development, nervous system development, neurogenesis and axonogenesis) to be over-represented (*p* < 0.05) (Fig. 3B). In this context many transcripts of interest were identified, for example, (1) *WNT7A,* encoding a factor previously implicated in the maintenance of adult neural stem cell self-renewal^34^; (2) *IGF1,* for which previous reports show levels in the SEZ to rapidly decrease over the course of aging^32^; (3) *IGF2*, which encodes a protein implicated in the maintenance of adult neurogenesis within the two forebrain neurogenic niches^35^ and (4) *TCF4*, which has previously been shown to regulate neuronal differentiation^36^. Analysis of upstream regulators via Ingenuity Pathway Analysis (IPA; Fig. 3C) further identified *EOMES* to be in this profile. *EOMES* mRNA encodes a factor expressed by a population of progenitor cells within the adult SEZ that generates glutamatergic olfactory bulb interneurons^37^. Supplementary Figure 1 reveals the relationship between the genes identified in this profile using STRING analysis^38^. We validated two of the transcripts identified in this profile, *DLX1* and *IGF1,* using a second, larger cohort of samples (see “Methods” section; this cohort only included 7 age groups due to limited tissue availability from cases in the toddler and school age group). *Dlx1* has previously been shown to be critical to the generation of telencephalic interneurons^39^. Consistent with our RNA sequencing data, qPCR revealed a sharp reduction in *DLX1* mRNA levels in neonate samples compared to children (*p* < 0.001), adolescents (*p* < 0.001), young adults (*p* < 0.001), adults (*p* < 0.001) and aged adults (*p* < 0.001; Fig. 3D). Similarly, qPCR confirmed a significant decline in *IFG1* expression in all age groups compared to neonates (Fig. 3E), further supporting previous observations^32^.

### Genes that increased gradually over the course of aging

**Figure 4:**
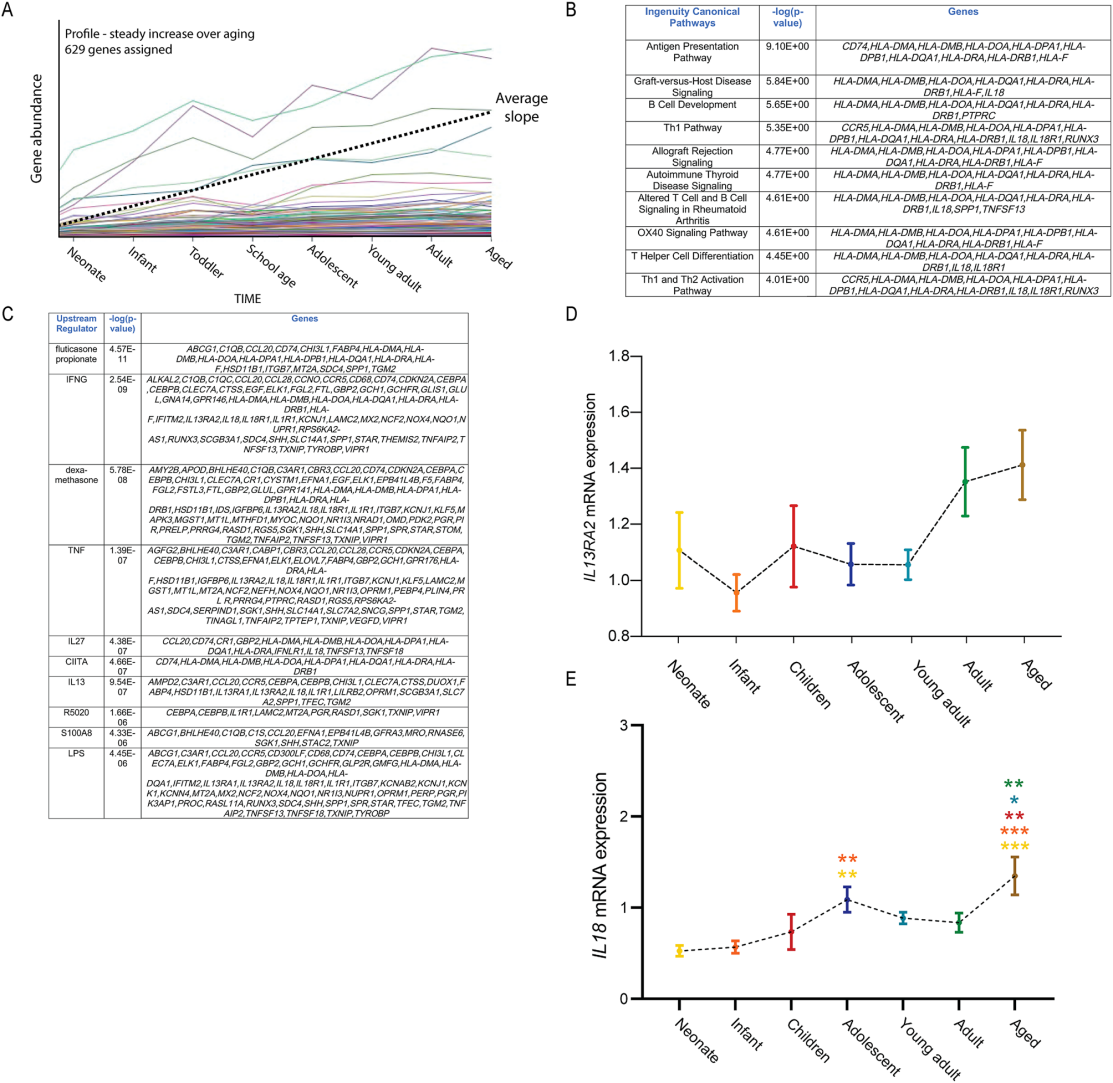
Transcripts whose expression rose steadily over the course of aging. (**A**) The second major profile that we identified related to genes whose expression increased steadily over the course of aging. There were 629 genes associated with this profile. Ingenuity analysis revealed many immune-associated pathways associated with this profile (**B**), as well as many immune-associated upstream regulators potentially linked to it (**C**). (**D**,**E**) qPCR analysis confirmed the steady rise in the expression of *IL13RA2* (**D**) and *IL18* (**E**). Colours for significance (***) correspond to the colour of the age group used in the comparison. *p < 0.05, **p < 0.01, ***p < 0.001; Analysis of variance, post hoc tests using LSD.

The second profile of interest reflected transcripts that displayed a steady increase in levels over time (Fig. 4A). There were 629 genes associated with this profile (Supplementary Table 5). IPA analysis of genes in this profile identified numerous pathways associated with the immune system, including regulation of T-cell and B-cell proliferation and activation, antigen presentation and cell adhesion (Fig. 4B). Moreover, IPA analysis of potential upstream regulators revealed other factors associated with immune activation, including IL27^40^ and IL13^41^ (Fig. 4C). Examples of genes found within this profile included nine of the 21 MHC protein complex elements (e.g. *HLA-DMA, HLA-DMB, HLA-DOA, HLA-DRA*), multiple fibronectin binding proteins (e.g. *cathepsin S, myocilin* and a *pleckstrin homology domain containing protein*), cytokines and their receptors (e.g. *IL18, IL1R1, IL13RA2, IL18R1*), components of the tumour necrosis factor pathway and a range of other immune system-associated genes (e.g. *CD74, SPP1, CCR5, TNFSF18*). Interestingly, there were also 18 transcripts implicated in the regulation of neurotransmitter levels, 12 genes previously assessed in microglia during Alzheimer’s disease^42^ and 37 transcripts associated with schizophrenia^43^ contained in this profile. A STRING analysis of the factors identified in this profile is shown in Supplementary Figure 2. We confirmed a non-significant increase over postnatal age for one of these factors, *IL13RA2*, using qPCR (Fig. 4D, p = 0.056). Moreover, we further validated a second target gene, *IL18* (Fig. 4E). The levels of this gene also gradually increased over the course of aging.

### Genes that increased in the aged SEZ

**Figure 5:**
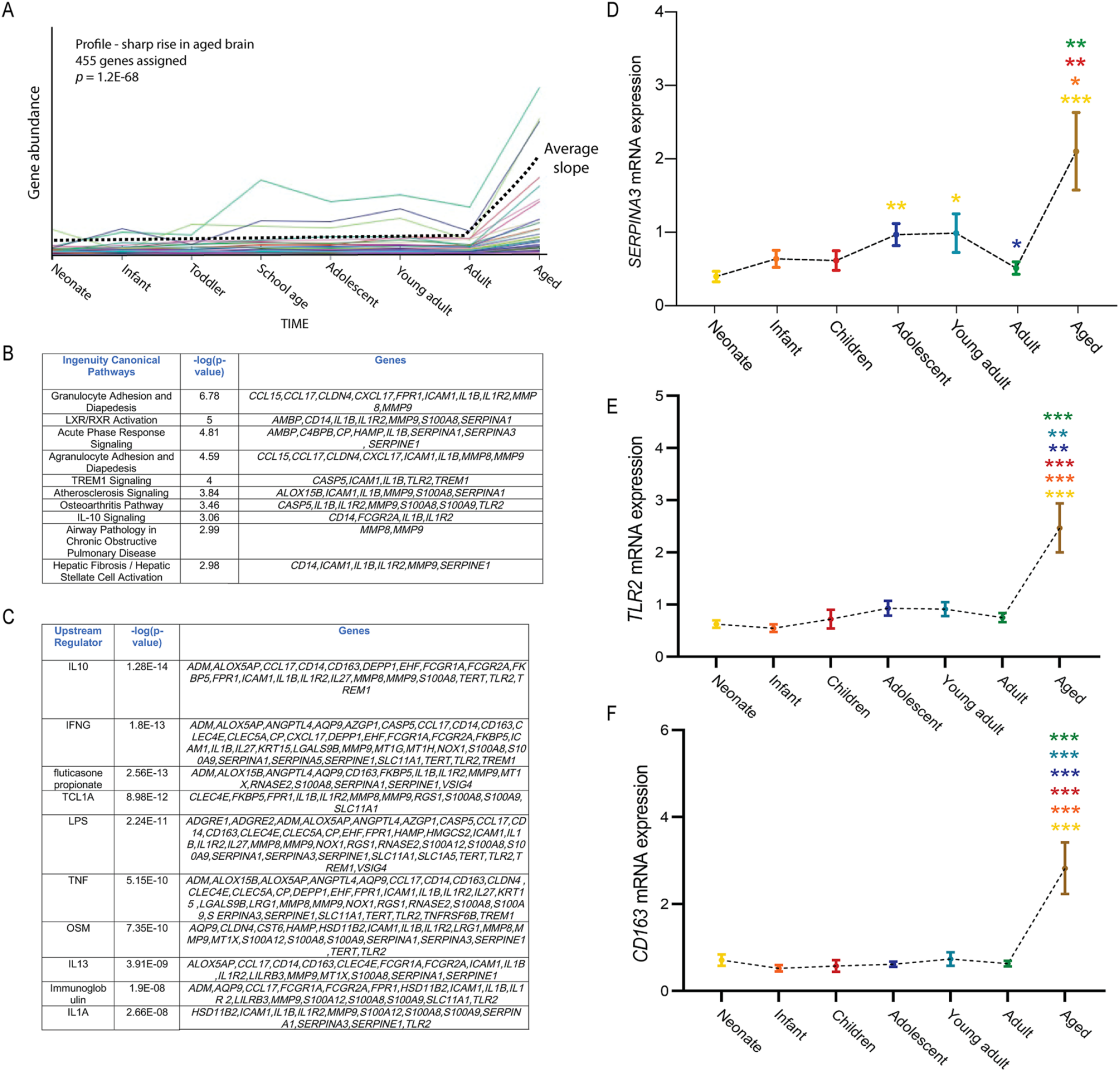
Transcripts that showed a sharp increase in expression within the aged SEZ. (**A**) The third major profile that we identified related to genes whose expression was relatively steady within the SEZ throughout life, until increasing sharply within the aged brain. There were 455 genes associated with this profile. Ingenuity analysis revealed many immune-associated pathways associated with this profile (**B**), as well as many immune-associated upstream regulators potentially linked to it (**C**). (**D**–**F**) The expression of *SERPINA3* (**D**), *TLR2* (**E**) and *CD163* (**F**) as measured by qPCR was consistent with our sequencing data, with the expression of these genes being significantly higher in the aged brain when compared to other age groups. Colours for significance (***) correspond to the colour of the age group used in the comparison. *p < 0.05, **p < 0.01, ***p < 0.001; Kruskal–Wallis H test or analysis of covariance, post hoc tests with Bonferroni or LSD.

Finally, the third profile of interest contained transcripts with no clear change in expression during the first 5 decades of life, but with a marked increase in the aged brain (Fig. 5A). In total, 455 genes were identified whose expression pattern over life followed this profile (Supplementary Table 6). Analysis of this cohort identified a mixed group of canonical pathways that included many immune-associated genes (e.g. *CXCL1*, *CCL15, CCL17, IL1B, IL1R2, CD14*) (Fig. 5B). The involvement of immune activation in this profile was also identified through the analysis of potential upstream regulators, which included IL10, IFNG, TNF and IL1A (Fig. 5C). Many other factors previously associated with inflammation, such as *CD163*^44^ and *TLR2*^45^ were also associated with this profile. As with the steadily aging profile, we revealed that this profile included members of the serpin family of protease inhibitors (e.g. *SERPINA1, SERPINA3, SERPINE3*), matrix metalloproteinases (e.g. *MMP8* and *MMP9*) and calcium-binding proteins (e.g. *S100A9* and *S100A12*). A STRING analysis of the factors identified in this profile is shown in Supplementary Figure 3. qPCR validation of three of the transcripts in this profile, *SERPINA3, TLR2* and *CD163,* confirmed the significant increase in expression of these factors in the aged SEZ in comparison to the adult SEZ (Fig. 5D–F), and also identified that *SERPINA3* mRNA is increased to a lesser degree in the adolescent and young adult compared to the neonate (*p* < 0.01and *p* < 0.05).

## Discussion

Here, we provide a novel resource to the research community, namely a transcriptomic dataset that describes the changes in gene expression occurring in the SEZ over the course of the entire human lifespan. The RNA sequencing data described here form part of a larger dataset we recently published that investigated changes occurring in the SEZ in patients with schizophrenia^14^. In this study, we provide evidence to support a decrease in the expression of markers for neurogenesis across the course of aging (e.g. *DCX* expression). Critically, we also revealed decreased expression of markers for neurogenesis within the SEZ of patients with schizophrenia and bipolar disorder^14^. These data provide novel insights into adult neurogenesis within the SEZ of patients with psychiatric disorders. However, they also provide a rich dataset that we leveraged here to better understand gene expression changes within the SEZ over the lifespan. Consistent with previous reports in humans, we revealed a significant number of genes whose expression dropped quickly after birth (Fig. 3), concomitant with the rapid decrease in neurogenesis that occurs within the SEZ in the first few years of life^9,14,17^. Once the increased levels of these transcripts associated with the early period of rapid neurogenesis appears to stabilize, we identified two additional and distinct profiles of age-related mRNA expression changes that appear to start, one with a gradual rise during aging, and one with a sharper rise at old age. Both of these profiles contain markers of neuroinflammation. Inflammation at a systemic level has also been shown to be a hallmark of aging^46^. These results suggest that multiple inflammatory processes may be present in the aging SEZ as they are in the body and other parts of the brain^47,48^. Collectively, these data are consistent with previous reports suggesting that immune activation increases over the course of adult life and into aging, both systemically^46^, and within the SEZ^31^.

Our findings also provide novel insights into the processes underpinning ongoing neurogenesis within the human SEZ. Previous reports both from mice^24^ and humans^13,17^ suggest that neurogenesis within the SEZ declines across the course of aging. Transcriptomic analysis revealed a sharp decline early in life in many genes associated with neurogenesis, including *IGF1, IGF2* and *WNT7A*, consistent with these findings. Indeed, a recent report using single-cell RNA sequencing of young versus old quiescent neural stem cells within the mouse SEZ highlighted the connection between increased expression of the WNT antagonist *Sfrp5* and decreased neurogenesis as a result of increased neural stem cell quiescence^49^. This work also highlights a potential difference in the rodent versus the human SEZ. Whereas neural stem cell numbers decrease within the mouse SEZ over the course of aging^24^, we have previously shown that transcript levels of the neural stem cell marker *GFAPD* remain stable in the human SEZ through aging^12,14^. Furthermore, our work identified a cohort of other genes whose expression follows a similar trajectory, therefore revealing factors that may contribute to the decline in neurogenesis, including *TCF4*^50^. One caveat to these findings is that the sequencing was performed on bulk tissue, and as such, it is not possible to determine which cell type(s) within the SEZ display altered gene expression for these factors. Looking ahead, replicating some of these analyses using single-cell RNA sequencing would provide an avenue to assess cell-type specific changes in gene expression with much more granularity. Indeed, recent single-cell sequencing studies performed in the adult mouse SEZ provide evidence to support our findings^51,52^. For example, the expression of DCX at both a mRNA and protein level decreases over the course of aging in the mouse SEZ^52^.

Interestingly, two of the three profiles that we discuss in detail contained a large proportion of genes linked to inflammatory pathways. These findings are consistent with previous reports linking elevated inflammation to reductions in adult neurogenesis^26,27,49^. Indeed, a recent single-cell transcriptomic analysis of young and old forebrain neurogenic niches in mice revealed significant T cell infiltration in the old niches, and these cells inhibit neural stem cell proliferation^53^. Likewise, another single-cell transcriptomic analysis of the mouse SEZ over the course of aging has also revealed that the aged SEZ is associated with a pro-inflammatory state^51^. In contrast, a recent study of the adult mouse SEZ using single-cell transcriptomic analysis revealed that transient inflammation induced by lipopolysaccharide culminated in the *increased* elevation of some neural stem cell genes, including *Gfap, Prom1* and *Id1*^54^*.* Whether such a response would also be evident in the aged mouse SEZ, and how this could potentially manifest within the SEZ over the many decades of human life, remain open questions. Intriguingly, studies in rodents using exercise to enhance adult neurogenesis have revealed that voluntary exercise can enhance adult neurogenesis in older mice, but only up to a certain age (18 months), after which exercise no longer had any beneficial effect on neurogenesis^55^. Although we do not know of the level of exercise that patients in the cohort performed, it is tempting to speculate that the significant increase in the genes in the third profile (genes with expression that increased in the aged SEZ) could contribute to this effect, potentially by contributing to a level of inflammation whose effects on neurogenesis can no longer be ameliorated by exercise. One way in which this could be investigated could be to use rodents, and to ectopically drive the expression of some of these genes within the neurogenic niche, using the rationale that this could lead to a premature reduction in SEZ-derived neurogenesis. Alternatively, aged mice could be assayed again in these exercise protocols using methods aimed at minimising inflammation, similar to earlier reports in the murine hippocampus^27^. Our finding that *SERPINA3* expression increased in the aged SEZ, together with other studies showing that this gene is highly upregulated in astrocytes in the dorsolateral prefrontal cortex of schizophrenia patients^56^, especially in those with elevated brain cytokines, suggests *SERPINA3* may be a potential marker of pathogenic brain inflammation.

Bioinformatic methods, as have been demonstrated in this study, can be powerful in detecting genes, pathways and upstream processes that may be targeted clinically to alleviate the deterioration of human brain function. In a clinical setting, these results also help point to key signalling pathways, such as the WNT and inflammatory pathways, that may be modulated through small molecule, antibody or antisense techniques in combination. Decreasing inflammatory cascades in the SEZ, combined with WNT pathway stimulation, could lead to increased neurogenic capacity in older age. These ideas are evident in age-related diseases, such as Alzheimer’s disease, in which modulation of these two pathways may be advantageous to patients^57,58^.

## Methods

### Human post-mortem brain samples

Data collection and molecular biology experiments for this study were conducted at the University of New South Wales under the ethics certificates HC16441 and HC16442, and were carried out in accordance with the Declaration of Helsinki. Fresh-frozen brain tissue was provided by the University of Maryland Brain and Tissue Bank (Baltimore, USA), New South Wales Brain Tissue Resource Centre (Sydney, Australia) and Sydney Brain Bank (Sydney, Australia). Details of the cohort demographics are provided in Supplementary Tables 1 and 2.

### Processing of brain tissue

Fresh-frozen tissue was removed from the anterior-third of the caudate nucleus at the level shown in photographs on pages 121–123 of the *Atlas of the Human Brain*^59^. Tissue was cut in the coronal plane into 12 × 60 μm sections and stored on wax paper. A scalpel was used to dissect the SEZ from the caudate nucleus in all 60 µm frozen tissue sections maintained on dry ice (∼30 mg tissue total). Luxol fast blue staining for myelin was performed for each case to facilitate accurate dissection of the SEZ. All tissue was stored at − 80 °C.

### RNA extraction and cDNA synthesis

Total RNA was extracted from SEZ tissue using TRIzol as per the manufacturer’s protocol (Thermo Fisher Scientific, Carlsbad, CA, USA). RNA concentration was assessed on the Nanodrop ND-1000 spectrophotometer (Thermo Fisher Scientific). RNA quality was assessed on the Agilent Technologies 2100 Bioanalyzer (Agilent Technologies, Santa Clara, CA, USA). cDNA was synthesised from 3 µg total RNA using SuperScript® First-Strand Synthesis kit IV and random hexamers following the manufacturer’s protocol (Thermo Fisher Scientific).

### Library preparation and RNA sequencing

RNA sequencing libraries were constructed using the TruSeq Stranded Total RNA Library Prep Gold kit as per the manufacturer’s protocol (Illumina, Inc., San Diego, CA, USA). Briefly, 1 μg of total RNA was used as input to the ribosomal RNA depletion followed by fragmentation and cDNA generation. The cDNA library was amplified using 12 PCR cycles. cDNA libraries were pooled and treated with Free Adapter Blocking Reagent (Illumina, Inc.) to ensure removal of free adapters. Quality was assessed after ribosomal RNA depletion and library preparation using Agilent Technologies 2100 Bioanalyzer (Agilent Technologies). In total 2 technical replicates of 5 biological replicates for each of the 8 different life-stages being studied were sequenced. Paired-end sequencing of 100 bp read lengths was conducted by the Ramaciotti Centre for Genomics (University of New South Wales, Sydney, Australia) using an S2 flowcell on the NovaSeq 6000 (Illumina, Inc.). A high sequencing depth of paired-end reads per sample was attained with most samples having 50 million sequenced read pairs and the lowest depth being just below 40 million.

### Data analysis

Demultiplexing was performed using bcltofastq2 version 2.20 (Illumina, Inc. California, United States). Illumina-specific adapter sequences were removed using the ILLUMINACLIP function of Trimmomatic version 0.36. FASTQ file quality was checked using FastQC version 0.11.5 (bioinformatics.babraham.ac.uk/projects/fastqc) and MultiQC before and after trimming reads based on quality scores with Trimmomatic version 0.36. The average Phred quality score (both overall and per position) across reads remained over 35 before and after trimming. Principal component analysis showed very high similarity of technical replicates. One control case presented as an extreme outlier in principal component analysis. Further investigation revealed a high frequency of duplicated reads (around 60%) and discrepant GC content (above 50%) in this sample, which was excluded from the dataset to preserve future analyses. STAR version 2.5.2a^60^ was used to align reads to the reference transcriptome from Genome Reference Consortium Human Build 38. Parameters for STAR alignment were kept as default values, except: length of overhang in junctions was set to 100, maximum number of loci a read is allowed to map was set to 1, maximum number of mismatches was made virtually unlimited, maximum ratio between mismatches and mapped read length was set to 0.1. RSEM version 1.2.30^61^ as used to calculate expected counts with parameters kept as default. On average, after trimming (which only removed 2–5 million reads), 20–30 million sequences reached a Phred score of 36 (very high quality) and 85–90% of the read pairs aligned uniquely to the transcriptome maintaining proper pairing.

Gene expression measured by RSEM as detailed above was assessed using STEM version 1.3.13. STEM is an acronym for Short Time-series Expression Miner, an algorithm supporting the clustering, comparison and visualization of gene expression data for short (e.g. 8 time points) time series that can differentiate between real and random patterns. STEM was used with no additional normalization step, requesting a maximum of 1000 expression profiles to be derived and every two consecutive time points in a given profile was allowed a maximum of 2 unit changes in any direction. These parameters were also altered to allow STEM to generate a limited number of profiles, which resulted in virtually the same profiles being classified as over-represented.

### Ingenuity pathway analysis

Ingenuity Pathway Analysis (content version: 49309495; release date: 30/08/2019) was used for canonical pathway and upstream regulator analysis. The webserver for STRING version 11^38^ was also consulted to assess the sets of genes in each of the three profiles of interest and to build gene networks based on reported physical and/or functional interactions and highlight enriched gene clusters.

### STRING analysis

String v.11.5^38^ was used to generate protein–protein interaction (PPI) networks from the genes in each profile of interest (gene lists are provided in Supplementary Tables 4–6). Interactions can be based on direct (physical contacts) or indirect (functional relationships) and cover associations supported by experimental evidence (e.g. experiments, co-expression, genomic location) and/or computational predictions (e.g. text-mining, databases). For each of the three profiles described here, a comprehensive network showing all annotated genes of the profile and linking those with interaction scores of at least 0.4 (medium confidence) is shown in Supplementary Figures 1–3. For all networks, functionally enriched classes of special interest were manually selected and coloured by String in the "analysis" tab of the webserver. All networks had PPI enrichment *p* values < 3.0e−12 and only classes with FDR < 0.05 for functional enrichment were selected for highlights.

### Quantitative reverse transcription polymerase chain reaction

Gene expression was measured on the BioMark™ HD system (Fluidigm, South San Francisco, CA, USA) based on 96.96 Dynamic Array™ integrated fluidic circuit (GE chip) using TaqMan Gene Expression Assays (Supplementary Table 7). Gene expression measurements were conducted by the Ramaciotti Centre for Genomics (University of New South Wales, Sydney, Australia) according to the manufacturer’s protocol. Relative quantities were determined from a seven-point standard curve of pooled cDNA. The ‘no template controls’ did not produce a signal for any mRNA examined. Five housekeeper genes, β-actin (*ACTB*), TATA-box binding protein (*TBP*), ubiquitin C (*UBC*), glyceraldehyde-3-phosphate dehydrogenase (*GAPDH*) and β-2-microglobulin were initially assessed in the SEZ. *ACTB*, *GAPDH* and *UBC* were used based on stable expression across age groups to calculate the normalising factor for gene expression (geometric mean, all *p* > 0.05, data not shown). The geometric mean did not significantly differ between age groups (*p* > 0.05, data not shown).

### Statistical analyses

Statistical analyses of gene expression measurements were performed with IBM SPSS Statistics version 26 (IBM, Armonk, NY, USA) and GraphPad Prism version 8 (GraphPad Software, La Jolla, CA, USA). Outliers were defined as values greater than two standard deviations from the group mean and were excluded from all analyses (1–5 individuals per target gene). Shapiro–Wilk tests were used to identify whether the distribution of data significantly differed from a normal distribution within each group. Pearson’s product-moment or Spearman’s rank correlations were conducted to assess the relationships between target gene expression and demographic variables and tissue quality factors for the cohort (Supplementary Table 3). For the statistical analysis of target gene expression, brain pH, RIN and PMI were included as covariates if a significant correlation was detected between a demographic variable and a target gene (Supplementary Table 3). Analyses of variance, analyses of covariance Kruskal–Wallis H test or Quade’s rank analyses of covariance were performed to assess age group differences for each target gene. Fisher’s least significant difference (LSD) or Bonferroni post hoc tests were used to determine specific differences between age groups. Relative gene expression was graphed as mean ± standard error of the mean. An α level of *p* ≤ 0.05 was considered statistically significant.

### Ethics declaration: approval for human experiments

This study was conducted at the University of New South Wales under the following ethics certificates (HC16441, HC16442), and was carried out in accordance with the Declaration of Helsinki. No further approvals from other institutional or licensing committees were required for this study.

## Supplementary Information

Supplementary Tables and Figures.

Supplementary Table 4.

Supplementary Table 5.

Supplementary Table 6.
