# Supplementary material for: Identifying gene expression profiles associated with neurogenesis and inflammation in the human subependymal zone from development through aging
Source: Sci Rep. 2022 Jan 7;12:40. doi: 10.1038/s41598-021-03976-4 (PMC8742079; doi:10.1038/s41598-021-03976-4)
Supplement: Supplementary file 1 — Supplementary Tables and Figures. [file 41598_2021_3976_MOESM1_ESM.docx]

**SUPPLEMENTARY TABLES**

**Supplementary table 1. Demographic details of the RNA sequencing cohort**

Supplementary table 1 provides the demographic details of the donors of the samples used in the RNA sequencing cohort.

|  | **Neonatal period** (n=5) | **Infancy**  (n=5) | **Toddler**  (n=5) | **School Age**  (n=5) | **Adolescence** (n=5) | **Young adulthood** (n=5) | **Adulthood**  (n=5) | **Aging**  (n=5) |
| --- | --- | --- | --- | --- | --- | --- | --- | --- |
| Age at death in years (range) | 0.16±0.06 (0.08-0.24) | 0.46±0.23 (0.25-0.82) | 2.00±0.35 (1.50-2.50) | 10.40±2.22 (8-12.5) | 16.2±1.15 (15-17.5) | 23.4±1.52 (21-25) | 39.90±5.86 (34-48.5) | 86.0±11.25 (74-103) |
| pH (range) | 6.60±0.12 (6.47-6.76) | 6.56±0.18 (6.26-6.71) | 6.64±0.19 (6.45-6.90) | 6.73±0.17 (6.44-6.85) | 6.73±0.20 (6.41-6.90) | 6.60±0.24 (6.18-6.77) | 6.44±0.39 (6.10-6.98) | 6.20±0.24 |
| PMI in hours (range) | 35.50±18.74 (17-56) | 24.7±10.18 (14-40.5) | 27.40±9.63 (20-44) | 14.20±5.93 (5-20) | 18.40±2.30 (16-21) | 25.20±11.40 (16-43.5) | 16.2±4.64 (12-23) | 30.60±11.25 (20-45) |
| RIN | 7.36±0.28 (6.9-7.6) | 7.58±0.54 (6.7-8.1) | 7.54±0.35 (7.0-7.9) | 7.50±0.55 (6.7-8.1) | 7.60±0.34 (7.2-8.0) | 7.80±0.75 (7.3-9.1) | 7.62±0.76 (6.8-8.3) | 7.32±0.45 (6.9-7.9) |
| Sex | 3M/2F | 3M/2F | 1M/4F | 1M/4F | 4M/1F | 4M/1F | 3M/2F | 3M/2F |
| Race | 5 African American | 4 African American, 1 Caucasian | 2 African American, 3 Caucasian | 1 African American, 4 Caucasian | 1 African American, 4 Caucasian | 5 African American | 4 African American, 1 Caucasian | 3 African American, 2 Caucasian |

**Supplementary table 2. Demographic details of the qPCR validation cohort**

Supplementary table 2 provides the demographic details of the donors of the samples used in the qPCR validation cohort.

|  | **Neonatal period** (n=10) | **Infancy**  (n=10) | **Childhood**  (n=10) | **Adolescence** (n=10) | **Young adulthood** (n=10) | **Adulthood**  (n=10) | **Aging**  (n=10) |
| --- | --- | --- | --- | --- | --- | --- | --- |
| Age at death in years (range) | 0.15±0.07 (0.01-0.24) | 0.58±0.28 (0.25-0.92) | 6.00±4.63 (1.50-12.50) | 16.35±1.47  (14-18) | 23.20±1.77  (20-25) | 41.10±4.86  (34-48.5) | 83.00±11.03 (68-103) |
| pH (range) | 6.48±0.19 (6.12-6.76) | 6.55±0.19 (6.26-6.87) | 6.61±0.26 (6.05-6.90) | 6.68±0.20 (6.27-6.90) | 6.54±0.24 (6.18-6.92) | 6.56±0.30 (6.10-6.98) | 6.32±0.23 (5.95-6.61) |
| PMI in hours (range) | 39.15±18.52 (17-64) | 24.60±10.80 (10-40.5) | 20.60±10.30 (5-44) | 19.20±6.86 (12-36.5) | 21.70±12.11 (7-43.5) | 19.30±5.29 (12-27) | 32.25±13.30 (18-58) |
| RIN | 6.46±1.23  (4.4-7.6) | 7.16±1.03  (5.1-8.4) | 7.18±1.40  (3.3-8.1) | 7.41±0.88  (5.4-8.6) | 7.70±0.73  (7.0-9.1) | 7.75±0.58  (6.8-8.3) | 7.36±0.49  (6.5-8.1) |
| Sex | 5M/ 5F | 7M/ 3F | 4M/ 6F | 9M/ 1F | 8M/ 2F | 8M/ 2F | 7M/ 3F |
| Race | 8 African American, 1 Caucasian, 1 Hispanic | 8 African American, 2 Caucasian | 3 African American, 7 Caucasian | 5 African American, 5 Caucasian | 7 African American, 3 Caucasian | 5 African American, 5 Caucasian | 5 African American, 5 Caucasian |

**Supplementary table 3. Relationships between target gene expression, demographic variables and tissue quality factors**

Supplementary table 3 details the relationships between demographic variables and tissue quality factors for the target genes validated in this study.

|  | Brain pH | | RIN | | PMI | |
| --- | --- | --- | --- | --- | --- | --- |
|  | r/ρ | *p* | r/ρ | *p* | r/ρ | *p* |
| *ACTB* | **0.325** | **0.006** | **0.563** | **<0.001** | 0.116 | 0.340 |
| *GAPDH* | **0.349** | **0.003** | **0.571** | **<0.001** | 0.034 | 0.782 |
| *UBC* | **0.266** | **0.026** | **0.502** | **<0.001** | 0.030 | 0.803 |
| Geomean | **0.314** | **0.008** | **0.582** | **<0.001** | 0.061 | 0.616 |
| *DLX1* | 0.159 | 0.205 | -0.030 | 0.814 | **0.255** | **0.040** |
| *IL13RA2* | -0.058 | 0.644 | 0.146 | 0.241 | -0.150 | 0.231 |
| *SERPINA3* | -0.071 | 0.568 | 0.028 | 0.822 | 0.000 | 0.999 |
| *CD163* | **-0.275** | **0.025** | 0.031 | 0.802 | 0.054 | 0.665 |
| *TLR2* | -0.028 | 0.821 | -0.205 | 0.096 | **0.254** | **0.038** |
| *IGF1* | 0.187 | 0.129 | -0.012 | 0.926 | **0.448** | **<0.001** |
| *IL18* | 0.090 | 0.461 | 0.235 | 0.052 | -0.211 | 0.081 |

**Supplementary table 4. List of genes in the profile showing a rapid decrease in expression after the neonatal period.**

Supplementary table 4 details those genes identified whose expression rapidly declines in the SEZ after the neonatal period.

**Supplementary table 5. List of genes in the profile showing steadily increasing expression during aging.**

Supplementary table 5 details those genes identified whose expression gradually increases in the SEZ over the course of aging.

**Supplementary table 6. List of genes in the profile showing a rapid increase in the aging SEZ neurogenic niche.**

Supplementary table 6 provides the list of genes identified whose expression rapidly increases in the aging SEZ compared to earlier time points.

**Supplementary table 7. TaqMan gene expression assays**

Supplementary table 7 provides the details of the TaqMan gene expression arrays used in the qPCR validation experiments.

| Gene name | Gene symbol | TaqMan assay ID | Interrogated Sequence |
| --- | --- | --- | --- |
| *β-actin* | *ACTB* | Hs99999903_m1 | NM_001101.3 |
| *Glyceraldehyde-3-phosphate dehydrogenase* | *GAPDH* | Hs99999905_m1 | NM_001289746.1, NM_002046.5 |
| *Ubiquitin C* | *UBC* | Hs00824723_m1 | NM_021009.6 |
| *Distal-less homeobox 1* | *DLX1* | Hs00698288_m1 | NM_178120.4 |
| *Interleukin 13 receptor subunit alpha 2* | *IL13RA2* | Hs00152924_m1 | NM_000640.2 |
| *Serpin family A member 3*  *Insulin-like growth factor 1*  *Interleukin 18*  *Toll-like receptor 2*  *Cluster of differentiation 163* | *SERPINA3*  *IGF1*  *IL18*  *TLR2*  *CD163* | Hs00153674_m1  Hs01547656_m1  Hs01038788_m1  Hs00610101_m1  Hs00174705_m1 | NM_001085.4  NM_000618.4, NM_001111283.2, NM_001111284.1  NM_001243211.1, NM_001562.3  NM_001318789.1, NM_001318790.1, NM_001318791.1, NM_001318793.1, NM_003264.4  NM_004244.5, NM_203416.3 |

**Supplementary Figure 1. Gene interaction network generated for genes whose expression decreased rapidly during childhood.**

**
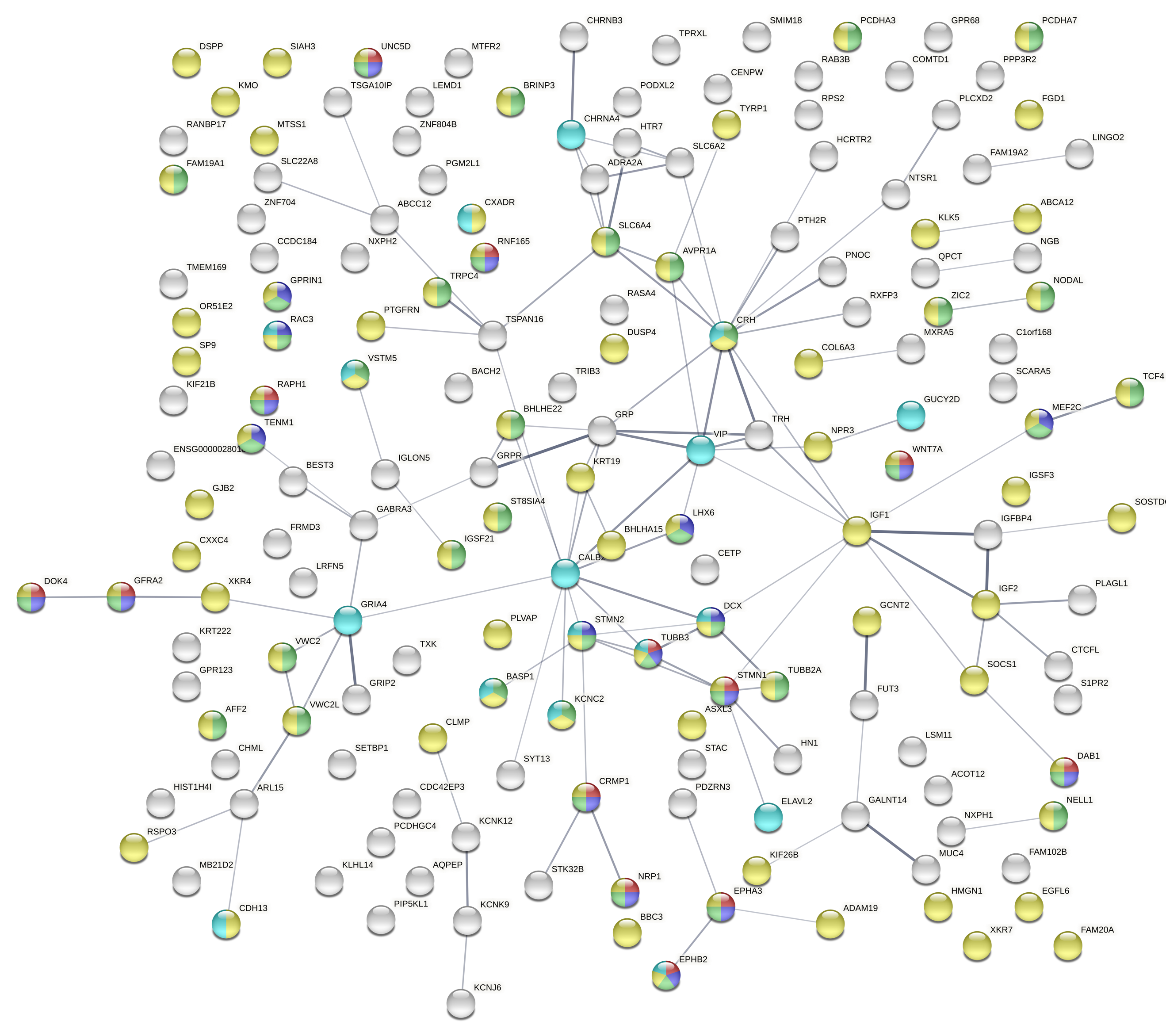
**

Gene interaction network generated with string-db for genes whose expression decreased rapidly during childhood (Supplementary Table 4). All identified genes with a minimum required interaction score (0.4 – medium confidence) are displayed. Enriched classes of Biological Processes of interest are coloured, and reflect the following processes: ‘axonogenesis’ (red), ‘neuron development’ (purple), ‘nervous system development’ (green) and ‘developmental processes’ (yellow). Also shown in aqua is the class of interest from the subcellular localization component of the network, ‘neuron projection’.

**Supplementary Figure 2. Gene interaction network generated for genes whose expression gradually increased over the course of aging.**


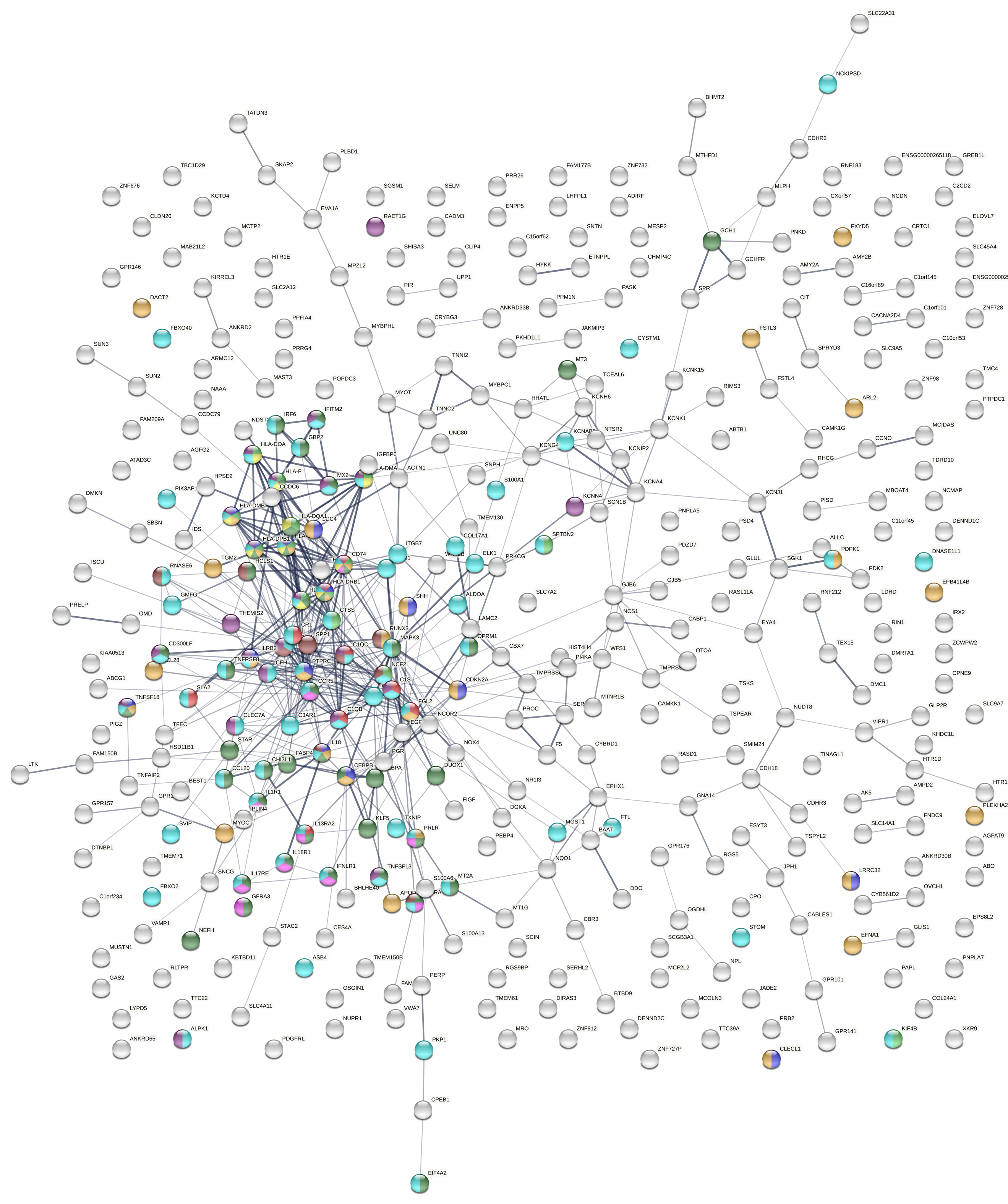


Gene interaction network generated with string-db for genes whose expression increased over the course of aging (Supplementary Table 5). All identified genes with a minimum required interaction score (0.4 – medium confidence) are displayed. Enriched classes of Biological Processes of interest are coloured, and reflect the following processes: ‘antigen processing and presentation of exogenous peptide’ (light green), ‘regulation of T cell proliferation’ (dark purple), ‘B cell mediated immunity’ (dark pink), ‘response to cytokine’ (dark green) and ‘regulation of cell adhesion’ (orange). For Annotated Keywords, genes involved in ‘immunity’ are shown in light purple. For reactome pathways, genes involved in ‘immune system’ are shown in aqua. For WikiPathways, genes involved in ‘TYROBP causal network in microglia’ are shown in brown. For Molecular Function, genes involved in ‘cytokine receptor activity’ are shown in light pink. For Cellular Component, genes involved in ‘MHC protein complex’ are shown in yellow.

**Supplementary Figure 3. Gene interaction network generated for genes whose expression increased in the aged SEZ.**

**
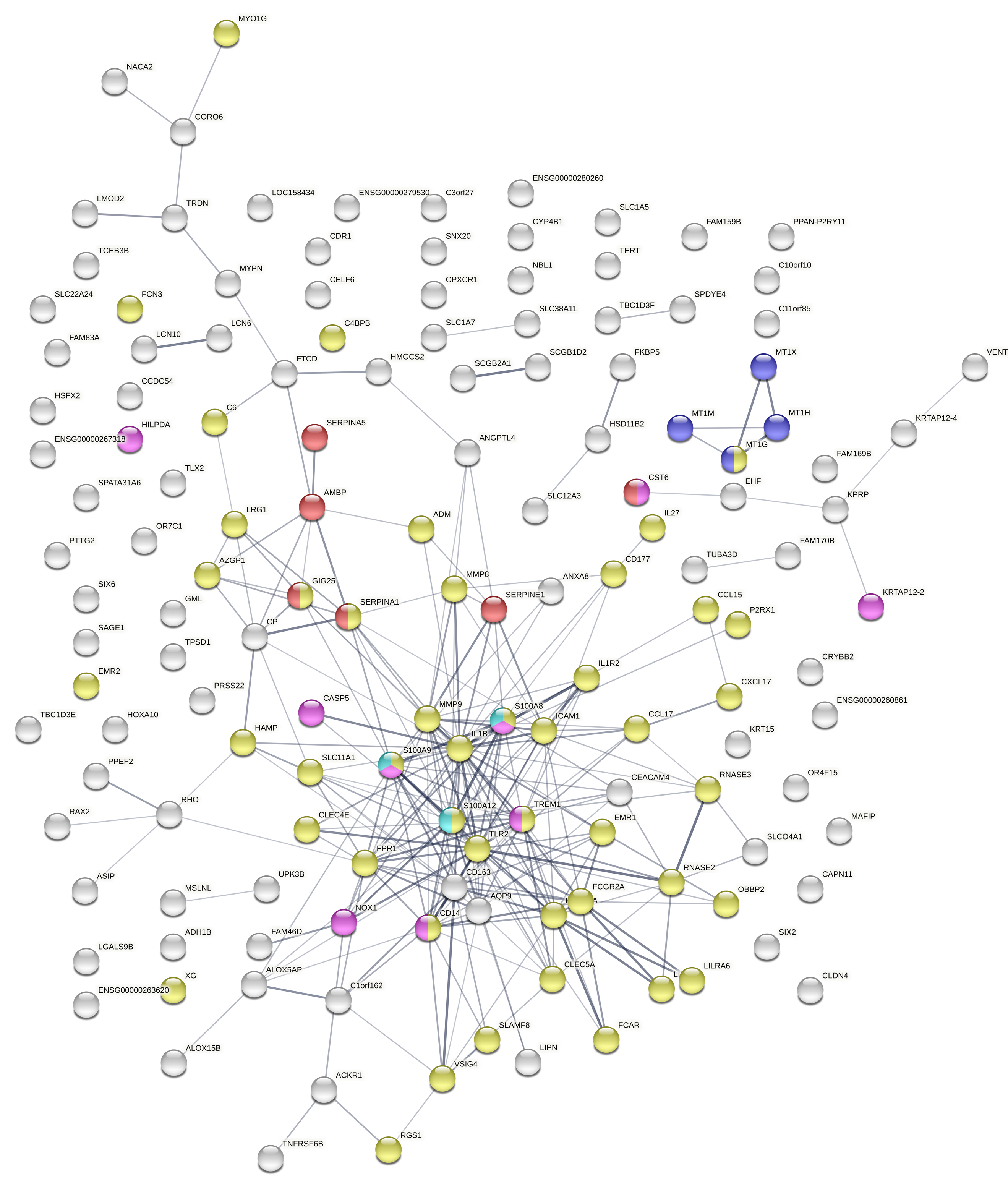
**

Gene interaction network generated with string-db for genes whose expression increased in the aged SEZ (Supplementary Table 6). All identified genes with a minimum required interaction score (0.4 – medium confidence) are displayed. Enriched classes of interest are coloured, and reflect the following biological processes: ‘immune system process’ (yellow). For Annotated Keywords, genes involved in ‘protease inhibitor’ are shown in dark pink. For reactome pathways, genes involved in ‘metallothioneins bind metals’ are shown in purple. For WikiPathways, genes involved in ‘vitamin D receptor pathway’ are shown in light pink. For Subcellular Localization, genes involved in ‘S100A8 complex’ are shown in aqua.
