# Supplementary material for: Identifying gene expression profiles associated with neurogenesis and inflammation in the human subependymal zone from development through aging
Source: Sci Rep. 2022 Jan 7;12:40. doi: 10.1038/s41598-021-03976-4 (PMC8742079; doi:10.1038/s41598-021-03976-4)
Supplement: Supplementary file 2 — Supplementary Table 4. [file 41598_2021_3976_MOESM2_ESM.pdf]

| | | |
|---|---|---|
| ENSG00000009694 | TENM1 | protein_coding |
| ENSG00000011677 | GABRA3 | protein_coding |
| ENSG00000017427 | IGF1 | protein_coding |
| ENSG00000019505 | SYT13 | protein_coding |
| ENSG00000043355 | ZIC2 | protein_coding |
| ENSG00000044524 | EPHA3 | protein_coding |
| ENSG00000072832 | CRMP1 | protein_coding |
| ENSG00000074966 | TXK | protein_coding |
| ENSG00000077279 | DCX | protein_coding |
| ENSG00000081189 | MEF2C | protein_coding |
| ENSG00000087237 | CETP | protein_coding |
| ENSG00000099250 | NRP1 | protein_coding |
| ENSG00000101188 | NTSR1 | protein_coding |
| ENSG00000101204 | CHRNA4 | protein_coding |
| ENSG00000101255 | TRIB3 | protein_coding |
| ENSG00000101825 | MXRA5 | protein_coding |
| ENSG00000102302 | FGD1 | protein_coding |
| ENSG00000103546 | SLC6A2 | protein_coding |
| ENSG00000104435 | STMN2 | protein_coding |
| ENSG00000105327 | BBC3 | protein_coding |
| ENSG00000105808 | RASA4 | protein_coding |
| ENSG00000106852 | LHX6 | protein_coding |
| ENSG00000107105 | ELAVL2 | protein_coding |
| ENSG00000107165 | TYRP1 | protein_coding |
| ENSG00000108576 | SLC6A4 | protein_coding |
| ENSG00000108950 | FAM20A | protein_coding |
| ENSG00000111846 | GCNT2 | protein_coding |
| ENSG00000112182 | BACH2 | protein_coding |
| ENSG00000113389 | NPR3 | protein_coding |
| ENSG00000113532 | ST8SIA4 | protein_coding |
| ENSG00000114631 | PODXL2 | protein_coding |
| ENSG00000115828 | QPCT | protein_coding |
| ENSG00000116852 | KIF21B | protein_coding |
| ENSG00000117009 | KMO | protein_coding |
| ENSG00000117154 | IGSF21 | protein_coding |
| ENSG00000117632 | STMN1 | protein_coding |
| ENSG00000118495 | PLAGL1 | protein_coding |
| ENSG00000119714 | GPR68 | protein_coding |
| ENSG00000120875 | DUSP4 | protein_coding |
| ENSG00000121440 | PDZRN3 | protein_coding |
| ENSG00000122584 | NXPH1 | protein_coding |
| ENSG00000125170 | DOK4 | protein_coding |
| ENSG00000126010 | GRPR | protein_coding |
| ENSG00000127325 | BEST3 | protein_coding |
| ENSG00000130167 | TSPAN16 | protein_coding |
| ENSG00000130300 | PLVAP | protein_coding |

| | | | |
|---|---|---|---|
| ENSG00000130600 | H19 | | processed_transcript |
| ENSG00000132518 | GUCY2D | | protein_coding |
| ENSG00000133107 | TRPC4 | | protein_coding |
| ENSG00000133216 | EPHB2 | | protein_coding |
| ENSG00000134247 | PTGFRN | | protein_coding |
| ENSG00000134443 | GRP | | protein_coding |
| ENSG00000135074 | ADAM19 | | protein_coding |
| ENSG00000137252 | HCRTR2 | | protein_coding |
| ENSG00000137267 | TUBB2A | | protein_coding |
| ENSG00000140798 | ABCC12 | | protein_coding |
| ENSG00000140945 | CDH13 | | protein_coding |
| ENSG00000140988 | RPS2 | | protein_coding |
| ENSG00000141431 | ASXL3 | | protein_coding |
| ENSG00000141622 | RNF165 | | protein_coding |
| ENSG00000141753 | IGFBP4 | | protein_coding |
| ENSG00000142549 | IGLON5 | | protein_coding |
| ENSG00000143061 | IGSF3 | | protein_coding |
| ENSG00000144227 | NXPH2 | | protein_coding |
| ENSG00000144407 | PTH2R | | protein_coding |
| ENSG00000144452 | ABCA12 | | protein_coding |
| ENSG00000144583 | | Mar-04 | protein_coding |
| ENSG00000144596 | GRIP2 | | protein_coding |
| ENSG00000144681 | STAC | | protein_coding |
| ENSG00000145113 | MUC4 | | protein_coding |
| ENSG00000146374 | RSPO3 | | protein_coding |
| ENSG00000146410 | MTFR2 | | protein_coding |
| ENSG00000146469 | VIP | | protein_coding |
| ENSG00000147432 | CHRNB3 | | protein_coding |
| ENSG00000147571 | CRH | | protein_coding |
| ENSG00000148680 | HTR7 | | protein_coding |
| ENSG00000149452 | SLC22A8 | | protein_coding |
| ENSG00000150594 | ADRA2A | | protein_coding |
| ENSG00000152217 | SETBP1 | | protein_coding |
| ENSG00000152578 | GRIA4 | | protein_coding |
| ENSG00000152591 | DSPP | | protein_coding |
| ENSG00000152953 | STK32B | | protein_coding |
| ENSG00000154639 | CXADR | | protein_coding |
| ENSG00000154764 | WNT7A | | protein_coding |
| ENSG00000155858 | LSM11 | | protein_coding |
| ENSG00000155966 | AFF2 | | protein_coding |
| ENSG00000156574 | NODAL | | protein_coding |
| ENSG00000156687 | UNC5D | | protein_coding |
| ENSG00000157542 | KCNJ6 | | protein_coding |
| ENSG00000158089 | GALNT14 | | protein_coding |
| ENSG00000162636 | FAM102B | | protein_coding |
| ENSG00000162670 | BRINP3 | | protein_coding |

| | | |
|---|---|---|
| ENSG00000162849 | KIF26B | protein_coding |
| ENSG00000163171 | CDC42EP3 | protein_coding |
| ENSG00000163359 | COL6A3 | protein_coding |
| ENSG00000163449 | TMEM169 | protein_coding |
| ENSG00000164684 | ZNF704 | protein_coding |
| ENSG00000165379 | LRFN5 | protein_coding |
| ENSG00000165434 | PGM2L1 | protein_coding |
| ENSG00000165474 | GJB2 | protein_coding |
| ENSG00000165553 | NGB | protein_coding |
| ENSG00000165644 | COMTD1 | protein_coding |
| ENSG00000165973 | NELL1 | protein_coding |
| ENSG00000166006 | KCNC2 | protein_coding |
| ENSG00000166148 | AVPR1A | protein_coding |
| ENSG00000166250 | CLMP | protein_coding |
| ENSG00000167103 | PIP5KL1 | protein_coding |
| ENSG00000167244 | IGF2 | protein_coding |
| ENSG00000167332 | OR51E2 | protein_coding |
| ENSG00000167754 | KLK5 | protein_coding |
| ENSG00000168079 | SCARA5 | protein_coding |
| ENSG00000168081 | PNOC | protein_coding |
| ENSG00000168546 | GFRA2 | protein_coding |
| ENSG00000168772 | CXXC4 | protein_coding |
| ENSG00000169213 | RAB3B | protein_coding |
| ENSG00000169258 | GPRIN1 | protein_coding |
| ENSG00000169427 | KCNK9 | protein_coding |
| ENSG00000169750 | RAC3 | protein_coding |
| ENSG00000170873 | MTSS1 | protein_coding |
| ENSG00000170893 | TRH | protein_coding |
| ENSG00000171124 | FUT3 | protein_coding |
| ENSG00000171243 | SOSTDC1 | protein_coding |
| ENSG00000171345 | KRT19 | protein_coding |
| ENSG00000172137 | CALB2 | protein_coding |
| ENSG00000172159 | FRMD3 | protein_coding |
| ENSG00000172497 | ACOT12 | protein_coding |
| ENSG00000172901 | AQPEP | protein_coding |
| ENSG00000173166 | RAPH1 | protein_coding |
| ENSG00000173406 | DAB1 | protein_coding |
| ENSG00000174453 | VWC2L | protein_coding |
| ENSG00000174482 | LINGO2 | protein_coding |
| ENSG00000175513 | TSGA10IP | protein_coding |
| ENSG00000176788 | BASP1 | protein_coding |
| ENSG00000177875 | CCDC184 | protein_coding |
| ENSG00000178162 | FAR2P2 | transcribed_unprocessed_pseudogene |
| ENSG00000178723 | GLULP4 | processed_pseudogene |
| ENSG00000180438 | TPRXL | protein_coding |
| ENSG00000180535 | BHLHA15 | protein_coding |

| | | |
|---|---|---|
| ENSG00000180611 | MB21D2 | protein_coding |
| ENSG00000180828 | BHLHE22 | protein_coding |
| ENSG00000182348 | ZNF804B | protein_coding |
| ENSG00000182631 | RXFP3 | protein_coding |
| ENSG00000183662 | FAM19A1 | protein_coding |
| ENSG00000184261 | KCNK12 | protein_coding |
| ENSG00000185305 | ARL15 | protein_coding |
| ENSG00000185338 | SOCS1 | protein_coding |
| ENSG00000186007 | LEMD1 | protein_coding |
| ENSG00000187889 | C1orf168 | protein_coding |
| ENSG00000188386 | PPP3R2 | protein_coding |
| ENSG00000188730 | VWC2 | protein_coding |
| ENSG00000189159 | HN1 | protein_coding |
| ENSG00000196628 | TCF4 | protein_coding |
| ENSG00000197177 | GPR123 | protein_coding |
| ENSG00000197705 | KLHL14 | protein_coding |
| ENSG00000198673 | FAM19A2 | protein_coding |
| ENSG00000198759 | EGFL6 | protein_coding |
| ENSG00000201643 | SNORA14A | snoRNA |
| ENSG00000203668 | CHML | protein_coding |
| ENSG00000203760 | CENPW | protein_coding |
| ENSG00000204764 | RANBP17 | protein_coding |
| ENSG00000204963 | PCDHA7 | protein_coding |
| ENSG00000205581 | HMGN1 | protein_coding |
| ENSG00000206579 | XKR4 | protein_coding |
| ENSG00000213553 | RPLP0P6 | processed_pseudogene |
| ENSG00000214376 | VSTM5 | protein_coding |
| ENSG00000215475 | SIAH3 | protein_coding |
| ENSG00000217236 | SP9 | protein_coding |
| ENSG00000223910 | ZNF32-AS3 | antisense |
| ENSG00000224020 | MIR181A2HG | antisense |
| ENSG00000224223 | VSTM2A-OT1 | sense_overlapping |
| ENSG00000225206 | MIR137HG | lincRNA |
| ENSG00000225649 | AC064875.2 | processed_transcript |
| ENSG00000226567 | LINC00606 | lincRNA |
| ENSG00000226683 | PWWP2AP1 | processed_pseudogene |
| ENSG00000227267 | RP11-305O4.2 | processed_pseudogene |
| ENSG00000227382 | EIF4A2P2 | processed_pseudogene |
| ENSG00000228663 | PSMD10P1 | processed_pseudogene |
| ENSG00000228817 | BACH1-IT2 | lincRNA |
| ENSG00000229153 | EPHA1-AS1 | antisense |
| ENSG00000229656 | RP11-462L8.1 | lincRNA |
| ENSG00000230397 | SPTLC1P1 | transcribed_processed_pseudogene |
| ENSG00000231011 | RP11-494I9.1 | processed_pseudogene |
| ENSG00000231121 | RP1-34H18.1 | lincRNA |
| ENSG00000231764 | DLX6-AS1 | antisense |

| | | |
|---|---|---|
| ENSG00000233093 | LINC00892 | processed_transcript |
| ENSG00000233860 | RP11-359D14.2 | antisense |
| ENSG00000235447 | TRAPPC13P1 | processed_pseudogene |
| ENSG00000236120 | RP11-733O18.1 | lincRNA |
| ENSG00000236166 | RP3-523C21.2 | lincRNA |
| ENSG00000237015 | CTA-984G1.5 | antisense |
| ENSG00000237596 | RP13-143G15.4 | antisense |
| ENSG00000239468 | RN7SL569P | misc_RNA |
| ENSG00000239959 | ENPP7P2 | unprocessed_pseudogene |
| ENSG00000240342 | RPS2P5 | processed_pseudogene |
| ENSG00000240801 | AC132217.4 | 3prime_overlapping_ncrna |
| ENSG00000240891 | PLCXD2 | protein_coding |
| ENSG00000240929 | HIST2H2BB | transcribed_unprocessed_pseudogene |
| ENSG00000241098 | FLJ46066 | lincRNA |
| ENSG00000241155 | ARHGAP31-AS1 | antisense |
| ENSG00000241933 | RP11-755B10.3 | lincRNA |
| ENSG00000242419 | PCDHGC4 | protein_coding |
| ENSG00000245156 | RP11-867G23.3 | lincRNA |
| ENSG00000248479 | RP11-807H7.2 | lincRNA |
| ENSG00000248708 | CTD-2316B1.2 | lincRNA |
| ENSG00000248927 | CTD-2334D19.1 | lincRNA |
| ENSG00000250896 | RNPS1P1 | processed_pseudogene |
| ENSG00000253213 | RP11-546B8.3 | processed_pseudogene |
| ENSG00000253389 | RP11-930P14.1 | antisense |
| ENSG00000253457 | SMIM18 | protein_coding |
| ENSG00000253966 | CTC-455F18.3 | processed_pseudogene |
| ENSG00000254305 | MRPL9P1 | transcribed_processed_pseudogene |
| ENSG00000255403 | AP000641.1 | processed_pseudogene |
| ENSG00000255408 | PCDHA3 | protein_coding |
| ENSG00000255547 | RPA2P3 | processed_pseudogene |
| ENSG00000256925 | GPR123-AS1 | antisense |
| ENSG00000257501 | RP11-1016B18.1 | transcribed_processed_pseudogene |
| ENSG00000257880 | RP11-769N19.2 | sense_intronic |
| ENSG00000258357 | RP11-778J16.2 | processed_pseudogene |
| ENSG00000258419 | RP11-588P7.1 | sense_intronic |
| ENSG00000258947 | TUBB3 | protein_coding |
| ENSG00000259871 | CTA-363E6.1 | sense_intronic |
| ENSG00000259957 | RP11-491F9.8 | lincRNA |
| ENSG00000260317 | RP11-48B3.4 | lincRNA |
| ENSG00000260412 | RP11-438B23.2 | sense_overlapping |
| ENSG00000260903 | XKR7 | protein_coding |
| ENSG00000261183 | RP11-532F12.5 | antisense |
| ENSG00000261888 | AC144831.1 | lincRNA |
| ENSG00000264058 | KRT222 | protein_coding |
| ENSG00000264885 | RP11-815I9.4 | sense_intronic |
| ENSG00000266588 | RP1-56K13.5 | lincRNA |

| | | |
|---|---|---|
| ENSG00000266923 | RP11-693J15.4 | antisense |
| ENSG00000267278 | MAP3K14-AS1 | antisense |
| ENSG00000267534 | S1PR2 | protein_coding |
| ENSG00000267764 | RP11-484L8.1 | antisense |
| ENSG00000267868 | RP11-120K24.3 | lincRNA |
| ENSG00000268051 | CTC-244M17.1 | sense_intronic |
| ENSG00000269867 | CTD-2583A14.8 | sense_intronic |
| ENSG00000270059 | RP11-88H12.2 | sense_intronic |
| ENSG00000270670 | RP11-248C1.3 | processed_pseudogene |
| ENSG00000271892 | CTD-2228A4.1 | lincRNA |
| ENSG00000271904 | CTC-498M16.4 | lincRNA |
| ENSG00000272627 | RP11-354E23.5 | sense_intronic |
| ENSG00000272689 | RP4-539M6.21 | sense_intronic |
| ENSG00000272717 | RP11-342I1.2 | antisense |
| ENSG00000273399 | RP11-408A13.3 | lincRNA |
| ENSG00000273447 | AC004067.5 | antisense |
| ENSG00000273786 | RP11-133K1.11 | sense_intronic |
| ENSG00000273853 | RP11-438D14.3 | sense_intronic |
| ENSG00000276180 | HIST1H4I | protein_coding |
| ENSG00000276261 | RP11-1060J15.9 | antisense |
| ENSG00000276449 | AC004076.5 | antisense |
| ENSG00000276740 | RP11-381O6.1 | sense_intronic |
| ENSG00000277182 | CTB-58E17.5 | antisense |
| ENSG00000277954 | RP11-679B19.1 | antisense |
| ENSG00000278532 | RP11-17E3.1 | lincRNA |
| ENSG00000278934 | CTD-2006M22.2 | TEC |
| ENSG00000279030 | RP11-212I21.3 | TEC |
| ENSG00000279206 | RP5-991G20.6 | lincRNA |
| ENSG00000279551 | RP11-588G21.1 | TEC |
| ENSG00000279601 | CTA-38K21.2 | TEC |
| ENSG00000279665 | RP11-507J18.5 | TEC |
| ENSG00000280048 | RP11-327J17.7 | TEC |
| ENSG00000280184 | AL023806.1 | protein_coding |
| ENSG00000280304 | CTD-2536I1.2 | TEC |
