# Supplementary material for: Identifying gene expression profiles associated with neurogenesis and inflammation in the human subependymal zone from development through aging
Source: Sci Rep. 2022 Jan 7;12:40. doi: 10.1038/s41598-021-03976-4 (PMC8742079; doi:10.1038/s41598-021-03976-4)
Supplement: Supplementary file 3 — Supplementary Table 5. [file 41598_2021_3976_MOESM3_ESM.pdf]

ENSG00000000971	CFH	protein_coding
ENSG00000003989	SLC7A2	protein_coding
ENSG00000004660	CAMKK1	protein_coding
ENSG00000005020	SKAP2	protein_coding
ENSG00000005882	PDK2	protein_coding
ENSG00000005981	ASB4	protein_coding
ENSG00000006555	TTC22	protein_coding
ENSG00000006747	SCIN	protein_coding
ENSG00000008118	CAMK1G	protein_coding
ENSG00000008394	MGST1	protein_coding
ENSG00000010282	HHATL	protein_coding
ENSG00000010404	IDS	protein_coding
ENSG00000011600	TYROBP	protein_coding
ENSG00000013563	DNASE1L1	protein_coding
ENSG00000019582	CD74	protein_coding
ENSG00000020129	NCDN	protein_coding
ENSG00000020633	RUNX3	protein_coding
ENSG00000022567	SLC45A4	protein_coding
ENSG00000034971	MYOC	protein_coding
ENSG00000043143	JADE2	protein_coding
ENSG00000047579	DTNBP1	protein_coding
ENSG00000053108	FSTL4	protein_coding
ENSG00000053524	MCF2L2	protein_coding
ENSG00000055732	MCOLN3	protein_coding
ENSG00000058085	LAMC2	protein_coding
ENSG00000062524	LTK	protein_coding
ENSG00000065325	GLP2R	protein_coding
ENSG00000065357	DGKA	protein_coding
ENSG00000065618	COL17A1	protein_coding
ENSG00000065923	SLC9A7	protein_coding
ENSG00000069424	KCNAB2	protein_coding
ENSG00000070404	FSTL3	protein_coding
ENSG00000071967	CYBRD1	protein_coding
ENSG00000072110	ACTN1	protein_coding
ENSG00000073331	ALPK1	protein_coding
ENSG00000074276	CDHR2	protein_coding
ENSG00000080200	CRYBG3	protein_coding
ENSG00000081237	PTPRC	protein_coding
ENSG00000081277	PKP1	protein_coding
ENSG00000082175	PGR	protein_coding
ENSG00000085831	TTC39A	protein_coding
ENSG00000086991	NOX4	protein_coding
ENSG00000087086	FTL	protein_coding
ENSG00000087250	MT3	protein_coding
ENSG00000087842	PIR	protein_coding
ENSG00000088340	FER1L4	unitary_pseudogene

ENSG00000088836	SLC4A11	protein_coding
ENSG00000089327	FXYD5	protein_coding
ENSG00000095203	EPB41L4B	protein_coding
ENSG00000095932	SMIM24	protein_coding
ENSG00000099308	MAST3	protein_coding
ENSG00000099937	SERPIND1	protein_coding
ENSG00000100181	TPTEP1	lincRNA
ENSG00000100206	DMC1	protein_coding
ENSG00000100242	SUN2	protein_coding
ENSG00000100285	NEFH	protein_coding
ENSG00000100307	CBX7	protein_coding
ENSG00000100341	PNPLA5	protein_coding
ENSG00000100714	MTHFD1	protein_coding
ENSG00000101082	SLA2	protein_coding
ENSG00000101298	SNPH	protein_coding
ENSG00000101470	TNNC2	protein_coding
ENSG00000101850	GPR143	protein_coding
ENSG00000102554	KLF5	protein_coding
ENSG00000102882	MAPK3	protein_coding
ENSG00000104213	PDGFRL	protein_coding
ENSG00000104369	JPH1	protein_coding
ENSG00000104783	KCNN4	protein_coding
ENSG00000105662	CRTC1	protein_coding
ENSG00000105711	SCN1B	protein_coding
ENSG00000105967	TFEC	protein_coding
ENSG00000106351	AGFG2	protein_coding
ENSG00000106976	DNM1	protein_coding
ENSG00000107130	NCS1	protein_coding
ENSG00000108091	CCDC6	protein_coding
ENSG00000108551	RASD1	protein_coding
ENSG00000109501	WFS1	protein_coding
ENSG00000112038	OPRM1	protein_coding
ENSG00000112319	EYA4	protein_coding
ENSG00000112378	PERP	protein_coding
ENSG00000112796	ENPP5	protein_coding
ENSG00000113296	THBS4	protein_coding
ENSG00000113494	PRLR	protein_coding
ENSG00000114626	ABTB1	protein_coding
ENSG00000114812	VIPR1	protein_coding
ENSG00000115009	CCL20	protein_coding
ENSG00000115295	CLIP4	protein_coding
ENSG00000115363	EVA1A	protein_coding
ENSG00000115594	IL1R1	protein_coding
ENSG00000115604	IL18R1	protein_coding
ENSG00000115648	MLPH	protein_coding
ENSG00000115687	PASK	protein_coding

ENSG00000115718	PROC	protein_coding
ENSG00000116096	SPR	protein_coding
ENSG00000116337	AMPD2	protein_coding
ENSG00000116661	FBXO2	protein_coding
ENSG00000116701	NCF2	protein_coding
ENSG00000117016	RIMS3	protein_coding
ENSG00000117594	HSD11B1	protein_coding
ENSG00000117595	IRF6	protein_coding
ENSG00000118515	SGK1	protein_coding
ENSG00000118785	SPP1	protein_coding
ENSG00000119227	PIGZ	protein_coding
ENSG00000120049	KCNIP2	protein_coding
ENSG00000120306	CYSTM1	protein_coding
ENSG00000120337	TNFSF18	protein_coding
ENSG00000120729	MYOT	protein_coding
ENSG00000120949	TNFRSF8	protein_coding
ENSG00000121316	PLBD1	protein_coding
ENSG00000121335	PRB2	protein_coding
ENSG00000121742	GJB6	protein_coding
ENSG00000122035	RASL11A	protein_coding
ENSG00000122966	CIT	protein_coding
ENSG00000123496	IL13RA2	protein_coding
ENSG00000124103	FAM209A	protein_coding
ENSG00000124145	SDC4	protein_coding
ENSG00000124249	KCNK15	protein_coding
ENSG00000125148	MT2A	protein_coding
ENSG00000125637	PSD4	protein_coding
ENSG00000126467	TSKS	protein_coding
ENSG00000126583	PRKCG	protein_coding
ENSG00000126767	ELK1	protein_coding
ENSG00000127083	OMD	protein_coding
ENSG00000127838	PNKD	protein_coding
ENSG00000127951	FGL2	protein_coding
ENSG00000128536	CDHR3	protein_coding
ENSG00000129226	CD68	protein_coding
ENSG00000130598	TNNI2	protein_coding
ENSG00000130653	PNPLA7	protein_coding
ENSG00000130755	GMFG	protein_coding
ENSG00000130775	THEMIS2	protein_coding
ENSG00000131042	LILRB2	protein_coding
ENSG00000131401	NAPSB	transcribed_unprocessed_pseud
ENSG00000131724	IL13RA1	protein_coding
ENSG00000131979	GCH1	protein_coding
ENSG00000132429	POPDC3	protein_coding
ENSG00000132840	BHMT2	protein_coding
ENSG00000133048	CHI3L1	protein_coding

ENSG00000133863	TEX15	protein_coding
ENSG00000134020	PEBP4	protein_coding
ENSG00000134042	MRO	protein_coding
ENSG00000134107	BHLHE40	protein_coding
ENSG00000134245	WNT2B	protein_coding
ENSG00000134508	CABLES1	protein_coding
ENSG00000134640	MTNR1B	protein_coding
ENSG00000135312	HTR1B	protein_coding
ENSG00000135378	PRRG4	protein_coding
ENSG00000135709	KIAA0513	protein_coding
ENSG00000135740	SLC9A5	protein_coding
ENSG00000135750	KCNK1	protein_coding
ENSG00000135821	GLUL	protein_coding
ENSG00000136003	ISCU	protein_coding
ENSG00000136881	BAAT	protein_coding
ENSG00000137507	LRRC32	protein_coding
ENSG00000137857	DUOX1	protein_coding
ENSG00000137880	GCHFR	protein_coding
ENSG00000138678	AGPAT9	protein_coding
ENSG00000138744	NAAA	protein_coding
ENSG00000138798	EGF	protein_coding
ENSG00000139190	VAMP1	protein_coding
ENSG00000139626	ITGB7	protein_coding
ENSG00000140519	RHCG	protein_coding
ENSG00000140563	MCTP2	protein_coding
ENSG00000140961	OSGIN1	protein_coding
ENSG00000140992	PDPK1	protein_coding
ENSG00000141449	GREB1L	protein_coding
ENSG00000141469	SLC14A1	protein_coding
ENSG00000141750	STAC2	protein_coding
ENSG00000142910	TINAGL1	protein_coding
ENSG00000143001	TMEM61	protein_coding
ENSG00000143248	RGS5	protein_coding
ENSG00000143257	NR1I3	protein_coding
ENSG00000143819	EPHX1	protein_coding
ENSG00000143847	PPFIA4	protein_coding
ENSG00000144406	UNC80	protein_coding
ENSG00000144410	CPO	protein_coding
ENSG00000144550	CPNE9	protein_coding
ENSG00000145063	AC062028.1	lincRNA
ENSG00000145526	CDH18	protein_coding
ENSG00000146013	GFRA3	protein_coding
ENSG00000146411	SLC2A12	protein_coding
ENSG00000147231	CXorf57	protein_coding
ENSG00000147465	STAR	protein_coding
ENSG00000147889	CDKN2A	protein_coding

ENSG00000148175	STOM	protein_coding
ENSG00000148671	ADIRF	protein_coding
ENSG00000148935	GAS2	protein_coding
ENSG00000149571	KIRREL3	protein_coding
ENSG00000149573	MPZL2	protein_coding
ENSG00000149925	ALDOA	protein_coding
ENSG00000150782	IL18	protein_coding
ENSG00000151062	CACNA2D4	protein_coding
ENSG00000151360	ALLC	protein_coding
ENSG00000151704	KCNJ1	protein_coding
ENSG00000151882	CCL28	protein_coding
ENSG00000152669	CCNO	protein_coding
ENSG00000153446	C16orf89	protein_coding
ENSG00000154027	AK5	protein_coding
ENSG00000155629	PIK3AP1	protein_coding
ENSG00000155719	OTOA	protein_coding
ENSG00000156049	GNA14	protein_coding
ENSG00000157343	ARMC12	protein_coding
ENSG00000157617	C2CD2	protein_coding
ENSG00000157782	CABP1	protein_coding
ENSG00000158079	PTPDC1	protein_coding
ENSG00000158220	ESYT3	protein_coding
ENSG00000159189	C1QC	protein_coding
ENSG00000159231	CBR3	protein_coding
ENSG00000159753	RLTPR	protein_coding
ENSG00000159871	LYPD5	protein_coding
ENSG00000160179	ABCG1	protein_coding
ENSG00000160183	TMPRSS3	protein_coding
ENSG00000160678	S100A1	protein_coding
ENSG00000160791	CCR5	protein_coding
ENSG00000161055	SCGB3A1	protein_coding
ENSG00000161132	XXbac-B444P24.10	unprocessed_pseudogene
ENSG00000161249	DMKN	protein_coding
ENSG00000161955	TNFSF13	protein_coding
ENSG00000162595	DIRAS3	protein_coding
ENSG00000162645	GBP2	protein_coding
ENSG00000162706	CADM3	protein_coding
ENSG00000162913	C1orf145	protein_coding
ENSG00000163131	CTSS	protein_coding
ENSG00000163239	TDRD10	protein_coding
ENSG00000163701	IL17RE	protein_coding
ENSG00000163833	FBXO40	protein_coding
ENSG00000164089	ETNPPL	protein_coding
ENSG00000164100	NDST3	protein_coding
ENSG00000164181	ELOVL7	protein_coding
ENSG00000164236	ANKRD33B	protein_coding

ENSG00000164488	DACT2	protein_coding
ENSG00000164690	SHH	protein_coding
ENSG00000164695	CHMP4C	protein_coding
ENSG00000164744	SUN3	protein_coding
ENSG00000164849	GPR146	protein_coding
ENSG00000165071	TMEM71	protein_coding
ENSG00000165188	RNF183	protein_coding
ENSG00000165197	FIGF	protein_coding
ENSG00000165370	GPR101	protein_coding
ENSG00000165887	ANKRD2	protein_coding
ENSG00000166073	GPR176	protein_coding
ENSG00000166448	TMEM130	protein_coding
ENSG00000166816	LDHD	protein_coding
ENSG00000167037	SGSM1	protein_coding
ENSG00000167608	TMC4	protein_coding
ENSG00000167676	PLIN4	protein_coding
ENSG00000167778	SPRYD3	protein_coding
ENSG00000167779	IGFBP6	protein_coding
ENSG00000167799	NUDT8	protein_coding
ENSG00000167995	BEST1	protein_coding
ENSG00000168405	CMAHP	unitary_pseudogene
ENSG00000168418	KCNG4	protein_coding
ENSG00000168830	HTR1E	protein_coding
ENSG00000169006	NTSR2	protein_coding
ENSG00000169242	EFNA1	protein_coding
ENSG00000169413	RNASE6	protein_coding
ENSG00000169499	PLEKHA2	protein_coding
ENSG00000170323	FABP4	protein_coding
ENSG00000170561	IRX2	protein_coding
ENSG00000171217	CLDN20	protein_coding
ENSG00000171502	COL24A1	protein_coding
ENSG00000171860	C3AR1	protein_coding
ENSG00000171889	MIR31HG	sense_overlapping
ENSG00000172216	CEBPB	protein_coding
ENSG00000172243	CLEC7A	protein_coding
ENSG00000172250	SERHL	processed_transcript
ENSG00000172568	FNDC9	protein_coding
ENSG00000172824	CES4A	protein_coding
ENSG00000172965	MIR4435-1HG	lincRNA
ENSG00000172987	HPSE2	protein_coding
ENSG00000173267	SNCG	protein_coding
ENSG00000173369	C1QB	protein_coding
ENSG00000173811	CCDC13-AS1	antisense
ENSG00000173826	KCNH6	protein_coding
ENSG00000173898	SPTBN2	protein_coding
ENSG00000174332	GLIS1	protein_coding

ENSG00000174370	C11orf45	protein_coding
ENSG00000174791	RIN1	protein_coding
ENSG00000175164	ABO	protein_coding
ENSG00000175894	TSPEAR	protein_coding
ENSG00000175984	DENND2C	protein_coding
ENSG00000176046	NUPR1	protein_coding
ENSG00000176399	DMRTA1	protein_coding
ENSG00000176595	KBTBD11	protein_coding
ENSG00000177106	EPS8L2	protein_coding
ENSG00000177669	MBOAT4	protein_coding
ENSG00000178222	RNF212	protein_coding
ENSG00000178343	SHISA3	protein_coding
ENSG00000178645	C10orf53	protein_coding
ENSG00000179083	FAM133A	protein_coding
ENSG00000179397	C1orf101	protein_coding
ENSG00000179546	HTR1D	protein_coding
ENSG00000180061	TMEM150B	protein_coding
ENSG00000180139	ACTA2-AS1	antisense
ENSG00000180229	HERC2P3	transcribed_unprocessed_pseud
ENSG00000180332	KCTD4	protein_coding
ENSG00000180353	HCLS1	protein_coding
ENSG00000180525	PRR26	protein_coding
ENSG00000180758	GPR157	protein_coding
ENSG00000180777	ANKRD30B	protein_coding
ENSG00000181019	NQO1	protein_coding
ENSG00000181541	MAB21L2	protein_coding
ENSG00000182255	KCNA4	protein_coding
ENSG00000182326	C1S	protein_coding
ENSG00000182487	NCF1B	transcribed_unprocessed_pseud
ENSG00000182508	LHFPL1	protein_coding
ENSG00000183486	MX2	protein_coding
ENSG00000183696	UPP1	protein_coding
ENSG00000183760	PAPL	protein_coding
ENSG00000183826	BTBD9	protein_coding
ENSG00000183935	HTR7P1	transcribed_processed_pseudog
ENSG00000184205	TSPYL2	protein_coding
ENSG00000184293	CLECL1	protein_coding
ENSG00000184454	NCMAP	protein_coding
ENSG00000185201	IFITM2	protein_coding
ENSG00000185215	TNFAIP2	protein_coding
ENSG00000185436	IFNLR1	protein_coding
ENSG00000186074	CD300LF	protein_coding
ENSG00000186326	RGS9BP	protein_coding
ENSG00000186777	ZNF732	protein_coding
ENSG00000186862	PDZD7	protein_coding
ENSG00000187037	GPR141	protein_coding

ENSG00000187045	TMPRSS6	protein_coding
ENSG00000187950	OVCH1	protein_coding
ENSG00000188095	MESP2	protein_coding
ENSG00000188266	HYKK	protein_coding
ENSG00000188277	C15orf62	protein_coding
ENSG00000188385	JAKMIP3	protein_coding
ENSG00000188783	PRELP	protein_coding
ENSG00000188817	SNTN	protein_coding
ENSG00000189001	SBSN	protein_coding
ENSG00000189058	APOD	protein_coding
ENSG00000189171	S100A13	protein_coding
ENSG00000189280	GJB5	protein_coding
ENSG00000189292	FAM150B	protein_coding
ENSG00000196091	MYBPC1	protein_coding
ENSG00000196109	ZNF676	protein_coding
ENSG00000196126	HLA-DRB1	protein_coding
ENSG00000196273	LINC00523	lincRNA
ENSG00000196735	HLA-DQA1	protein_coding
ENSG00000197360	ZNF98	protein_coding
ENSG00000197444	OGDHL	protein_coding
ENSG00000197503	LINC00477	lincRNA
ENSG00000197520	FAM177B	protein_coding
ENSG00000197837	HIST4H4	protein_coding
ENSG00000197934	CYYR1-AS1	antisense
ENSG00000197956	S100A6	protein_coding
ENSG00000198168	SVIP	protein_coding
ENSG00000198734	F5	protein_coding
ENSG00000198832	SELM	protein_coding
ENSG00000198959	TGM2	protein_coding
ENSG00000199363	SNORA63	snoRNA
ENSG00000203280	CTA-221G9.12	antisense
ENSG00000203705	TATDN3	protein_coding
ENSG00000203709	C1orf132	lincRNA
ENSG00000203710	CR1	protein_coding
ENSG00000203722	RAET1G	protein_coding
ENSG00000203797	DDO	protein_coding
ENSG00000203999	LINC01270	lincRNA
ENSG00000204071	TCEAL6	protein_coding
ENSG00000204252	HLA-DOA	protein_coding
ENSG00000204257	HLA-DMA	protein_coding
ENSG00000204287	HLA-DRA	protein_coding
ENSG00000204396	VWA7	protein_coding
ENSG00000204642	HLA-F	protein_coding
ENSG00000205038	PKHD1L1	protein_coding
ENSG00000205744	DENND1C	protein_coding
ENSG00000206195	DUXAP8	processed_transcript

ENSG00000206559	ZCWPW2	protein_coding
ENSG00000208839	SNORA35	snoRNA
ENSG00000213465	ARL2	protein_coding
ENSG00000213642	RP11-561N12.5	processed_pseudogene
ENSG00000213672	NCKIPSD	protein_coding
ENSG00000213889	PPM1N	protein_coding
ENSG00000214575	CPEB1	protein_coding
ENSG00000214652	ZNF727	protein_coding
ENSG00000215559	ANKRD20A11P	transcribed_unprocessed_pseud
ENSG00000215915	ATAD3C	protein_coding
ENSG00000216316	RP3-354N19.3	processed_pseudogene
ENSG00000216863	LY86-AS1	antisense
ENSG00000218424	NDUFS5P1	processed_pseudogene
ENSG00000221947	XKR9	protein_coding
ENSG00000221986	MYBPHL	protein_coding
ENSG00000222041	LINC00152	lincRNA
ENSG00000223485	RP11-417E7.1	lincRNA
ENSG00000223558	TRIM60P17	processed_pseudogene
ENSG00000223559	RP11-700P18.1	processed_pseudogene
ENSG00000223564	CYP4F32P	unprocessed_pseudogene
ENSG00000223865	HLA-DPB1	protein_coding
ENSG00000224116	INHBA-AS1	antisense
ENSG00000224222	RP11-262I2.2	lincRNA
ENSG00000224271	RP11-191L9.4	lincRNA
ENSG00000224441	AC068831.3	antisense
ENSG00000224577	LINC01117	lincRNA
ENSG00000224668	IPO8P1	processed_pseudogene
ENSG00000224689	ZNF812	protein_coding
ENSG00000224796	RPL32P1	processed_pseudogene
ENSG00000224950	RP5-1086K13.1	lincRNA
ENSG00000225110	LL0XNC01-16G2.1	unprocessed_pseudogene
ENSG00000225140	RP11-809C18.3	antisense
ENSG00000225285	RP4-758J18.10	lincRNA
ENSG00000225331	AP001055.6	lincRNA
ENSG00000225342	AC079630.4	antisense
ENSG00000225518	RP11-396C23.2	lincRNA
ENSG00000225778	PROSER2-AS1	antisense
ENSG00000226287	TMEM191A	processed_transcript
ENSG00000226578	RP11-258F22.1	lincRNA
ENSG00000226581	RP11-340I6.8	lincRNA
ENSG00000226608	FTLP3	processed_pseudogene
ENSG00000226650	KIF4B	protein_coding
ENSG00000227265	AC073464.4	processed_pseudogene
ENSG00000227392	HPN-AS1	antisense
ENSG00000227455	RP11-300M24.1	lincRNA
ENSG00000227646	STEAP2-AS1	processed_transcript

ENSG00000227835	CARM1P1	transcribed_unprocessed_pseud
ENSG00000227848	SUCLA2-AS1	antisense
ENSG00000227855	DPY19L2P3	transcribed_unprocessed_pseud
ENSG00000227868	C1orf234	protein_coding
ENSG00000227910	RP11-73B2.6	transcribed_processed_pseudog
ENSG00000228203	RNF144A-AS1	processed_transcript
ENSG00000228232	GAPDHP1	processed_pseudogene
ENSG00000228314	CYP4F29P	transcribed_unprocessed_pseud
ENSG00000228541	AC093159.1	lincRNA
ENSG00000228704	RP11-138M12.1	lincRNA
ENSG00000229081	LINC01165	antisense
ENSG00000229089	ANKRD20A8P	transcribed_unprocessed_pseud
ENSG00000229116	RP11-20J15.3	lincRNA
ENSG00000229321	AC008269.2	lincRNA
ENSG00000229427	ANKRD26P4	processed_pseudogene
ENSG00000229570	GAPDHP58	processed_pseudogene
ENSG00000230317	LINC01284	lincRNA
ENSG00000230965	SNX18P13	processed_pseudogene
ENSG00000231133	HAR1B	lincRNA
ENSG00000231367	AC016995.3	lincRNA
ENSG00000231389	HLA-DPA1	protein_coding
ENSG00000231616	RP11-575L7.4	antisense
ENSG00000231654	RPS6KA2-AS1	antisense
ENSG00000231683	RP1-27K12.2	lincRNA
ENSG00000231721	LINC-PINT	antisense
ENSG00000231826	AC016735.2	lincRNA
ENSG00000231995	RP11-111F5.2	processed_pseudogene
ENSG00000232006	AC005537.2	lincRNA
ENSG00000232352	SEMA3B-AS1	antisense
ENSG00000232386	RP11-66B24.2	lincRNA
ENSG00000232406	RP11-234K24.3	antisense
ENSG00000232514	RP11-329A14.2	processed_pseudogene
ENSG00000232656	IDI2-AS1	antisense
ENSG00000232706	NUTM2HP	unprocessed_pseudogene
ENSG00000232837	AF064858.7	lincRNA
ENSG00000233423	RP4-736H5.3	lincRNA
ENSG00000233435	AGGF1P2	processed_pseudogene
ENSG00000233593	RP4-665J23.1	lincRNA
ENSG00000233684	AC079779.6	lincRNA
ENSG00000233725	LINC00284	lincRNA
ENSG00000233820	RP11-535M15.2	transcribed_processed_pseudog
ENSG00000233878	AC073133.1	lincRNA
ENSG00000233885	YEATS2-AS1	antisense
ENSG00000234056	LINC00463	lincRNA
ENSG00000234076	TPRG1-AS1	lincRNA
ENSG00000234231	AC093616.4	unprocessed_pseudogene

ENSG00000234336	JAZF1-AS1	antisense
ENSG00000234602	MCIDAS	protein_coding
ENSG00000234840	LINC01239	lincRNA
ENSG00000235098	ANKRD65	protein_coding
ENSG00000235310	GXYLT1P6	processed_pseudogene
ENSG00000235595	GAPDHP23	processed_pseudogene
ENSG00000235831	BHLHE40-AS1	antisense
ENSG00000235884	LINC00941	lincRNA
ENSG00000236240	GPC5-IT1	sense_intronic
ENSG00000236451	AC067956.1	lincRNA
ENSG00000236700	LINC01010	lincRNA
ENSG00000236882	LINC01554	lincRNA
ENSG00000237595	RP11-112L6.3	lincRNA
ENSG00000237879	LINC00398	lincRNA
ENSG00000237949	LINC00844	lincRNA
ENSG00000240038	AMY2B	protein_coding
ENSG00000240271	RP11-200A13.2	processed_pseudogene
ENSG00000240405	LINC01212	lincRNA
ENSG00000240521	RP11-680B3.2	antisense
ENSG00000240912	RP11-274J15.2	processed_pseudogene
ENSG00000241231	RP11-275H4.1	lincRNA
ENSG00000241489	IDS	protein_coding
ENSG00000241717	VWFP1	transcribed_unprocessed_pseud
ENSG00000241878	PISD	protein_coding
ENSG00000241956	CTC-340A15.2	antisense
ENSG00000241973	PI4KA	protein_coding
ENSG00000242103	RP11-10G15.4	processed_pseudogene
ENSG00000242574	HLA-DMB	protein_coding
ENSG00000242768	RP11-556G22.1	processed_pseudogene
ENSG00000243480	AMY2A	protein_coding
ENSG00000243806	RPL7P18	processed_pseudogene
ENSG00000244089	HMGB1P30	processed_pseudogene
ENSG00000245848	CEBPA	protein_coding
ENSG00000245958	RP11-33B1.1	transcribed_unprocessed_pseud
ENSG00000246174	KCTD21-AS1	antisense
ENSG00000246225	RP11-17A1.3	antisense
ENSG00000246331	RP11-77I22.2	lincRNA
ENSG00000246430	LINC00968	lincRNA
ENSG00000246448	RP13-578N3.3	antisense
ENSG00000247011	RP11-700H6.1	lincRNA
ENSG00000247157	LINC01252	lincRNA
ENSG00000247287	RP11-902B17.1	antisense
ENSG00000248283	CCNL2P1	processed_pseudogene
ENSG00000248466	RP11-640B6.1	processed_pseudogene
ENSG00000248698	LINC01085	lincRNA
ENSG00000248994	RP11-259O2.1	lincRNA

ENSG00000249006	RP11-317B7.2	processed_pseudogene
ENSG00000249577	CTD-2195M15.1	processed_pseudogene
ENSG00000249604	RP11-286E11.2	antisense
ENSG00000249781	CTD-2143L24.1	lincRNA
ENSG00000249961	CCDC79	protein_coding
ENSG00000249996	RP11-359P5.1	antisense
ENSG00000250060	RP11-332J15.2	lincRNA
ENSG00000250125	RP11-707A18.1	lincRNA
ENSG00000250293	CRYZP2	processed_pseudogene
ENSG00000250770	RP5-1063M23.1	lincRNA
ENSG00000250878	METTL21EP	transcribed_unprocessed_pseud
ENSG00000251165	F11-AS1	antisense
ENSG00000251365	RP11-332J15.3	lincRNA
ENSG00000251372	LINC00499	lincRNA
ENSG00000251429	RP11-597D13.7	transcribed_processed_pseudog
ENSG00000251556	RP11-118M9.3	antisense
ENSG00000253394	LINC00534	lincRNA
ENSG00000253521	HPYR1	lincRNA
ENSG00000253647	CTD-2270F17.1	antisense
ENSG00000253693	CTC-535M15.2	lincRNA
ENSG00000253946	CTC-425K20.1	processed_pseudogene
ENSG00000253973	RP11-467K18.2	antisense
ENSG00000254211	LINC01485	lincRNA
ENSG00000254241	RP11-946L20.1	processed_pseudogene
ENSG00000254507	RP11-481A20.10	transcribed_processed_pseudog
ENSG00000254781	GVINP2	processed_pseudogene
ENSG00000254921	RP11-236J17.6	antisense
ENSG00000255020	AF131216.5	antisense
ENSG00000255120	OVOL1-AS1	antisense
ENSG00000255136	CTD-2562J17.4	lincRNA
ENSG00000255190	TRIM51DP	processed_pseudogene
ENSG00000255292	AP002884.2	protein_coding
ENSG00000255460	ZDHHC20P3	processed_pseudogene
ENSG00000255621	RP11-377D9.3	lincRNA
ENSG00000256050	RP11-982M15.6	lincRNA
ENSG00000256980	KHDC1L	protein_coding
ENSG00000257913	RP11-386G11.5	antisense
ENSG00000258122	RP11-61A14.1	antisense
ENSG00000258404	RP11-1029J19.5	lincRNA
ENSG00000258414	RP11-356O9.1	lincRNA
ENSG00000258472	RP11-192H23.4	protein_coding
ENSG00000258736	RP11-982M15.7	lincRNA
ENSG00000259017	RP11-561B11.6	lincRNA
ENSG00000259460	RP11-128A17.1	antisense
ENSG00000259583	RP11-66B24.4	antisense
ENSG00000259744	RP11-138H8.6	sense_intronic

ENSG00000259803	SLC22A31	protein_coding
ENSG00000259848	AC097374.2	transcribed_unprocessed_pseud
ENSG00000260186	RP11-481J2.2	lincRNA
ENSG00000260274	RP11-817O13.8	lincRNA
ENSG00000260418	RP3-406A7.7	lincRNA
ENSG00000260439	LMF1-AS1	antisense
ENSG00000260515	CTD-2199O4.3	lincRNA
ENSG00000260549	MT1L	unitary_pseudogene
ENSG00000260599	CTC-457E21.1	sense_intronic
ENSG00000260725	AC005307.1	lincRNA
ENSG00000260782	RP11-480G7.2	processed_pseudogene
ENSG00000260896	RP11-314O13.1	lincRNA
ENSG00000261033	RP11-209D14.2	antisense
ENSG00000261114	RP11-325K4.2	sense_intronic
ENSG00000261340	RP11-215H22.1	lincRNA
ENSG00000261399	LA16c-329F2.1	antisense
ENSG00000261617	RP11-243A14.1	lincRNA
ENSG00000261710	RP11-953B20.1	lincRNA
ENSG00000264617	AC144838.3	unprocessed_pseudogene
ENSG00000264707	L3MBTL4-AS1	lincRNA
ENSG00000265118	CTD-2370N5.3	protein_coding
ENSG00000265972	TXNIP	protein_coding
ENSG00000266248	CTC-525D6.2	antisense
ENSG00000266733	TBC1D29	protein_coding
ENSG00000267014	LINC01532	lincRNA
ENSG00000267191	RP11-15A1.2	antisense
ENSG00000267243	AC005307.3	lincRNA
ENSG00000268055	AC067969.2	antisense
ENSG00000268272	VN1R78P	unprocessed_pseudogene
ENSG00000269067	ZNF728	protein_coding
ENSG00000269345	VN1R85P	unprocessed_pseudogene
ENSG00000269935	RP11-482M8.3	antisense
ENSG00000269956	MKNK1-AS1	antisense
ENSG00000270969	CTD-2355J17.2	processed_pseudogene
ENSG00000271204	RP11-138A9.1	lincRNA
ENSG00000271434	RP13-238F13.3	processed_transcript
ENSG00000271858	CYB561D2	antisense
ENSG00000271947	RP11-439M11.1	lincRNA
ENSG00000272002	RP11-557L19.1	lincRNA
ENSG00000272112	CTB-113P19.5	lincRNA
ENSG00000272356	RP5-1112D6.8	antisense
ENSG00000272573	MUSTN1	protein_coding
ENSG00000272647	GS1-259H13.10	protein_coding
ENSG00000272808	RP11-66B24.7	processed_transcript
ENSG00000273024	INTS4P2	transcribed_unprocessed_pseud
ENSG00000273100	RP11-302L19.3	lincRNA

ENSG00000273305	RP11-440D17.4	lincRNA
ENSG00000273654	CTB-52I2.4	transcribed_processed_pseudog
ENSG00000274001	RP11-5G9.5	sense_intronic
ENSG00000274002	RP11-66N24.6	lincRNA
ENSG00000274292	RP11-347I19.7	lincRNA
ENSG00000275149	RP11-427J23.1	lincRNA
ENSG00000275160	RP11-187C18.3	processed_pseudogene
ENSG00000275350	RP11-329N22.2	processed_pseudogene
ENSG00000275759	RP11-131L12.3	lincRNA
ENSG00000276462	BX255923.3	lincRNA
ENSG00000276934	RP11-231E4.5	sense_intronic
ENSG00000277440	RP11-295D4.5	sense_intronic
ENSG00000277449	CEBPB-AS1	antisense
ENSG00000277587	CTD-3116E22.8	lincRNA
ENSG00000277734	TRAC	TR_C_gene
ENSG00000277895	RP11-114F3.4	antisense
ENSG00000278445	RP11-255P5.3	lincRNA
ENSG00000278703	RP11-706P11.2	lincRNA
ENSG00000279042	RP11-805F19.3	TEC
ENSG00000279137	RP11-205K6.3	TEC
ENSG00000279191	RP11-803D5.1	TEC
ENSG00000279205	RP11-632P5.1	TEC
ENSG00000279455	RP11-495K9.10	TEC
ENSG00000279692	RP11-1055B8.1	TEC
ENSG00000279720	bP-2171C21.6	processed_pseudogene
ENSG00000279853	RP5-844F9.1	TEC
ENSG00000280399	RP11-80P20.3	TEC
ENSG00000280439	RP4-568B10.1	TEC
ENSG00000280650	KCNIP4-IT1	lincRNA
ENSG00000280837	CPS1-IT1	sense_intronic
ENSG00000281883	RP11-65B7.2	protein_coding

ogene

ogene

ogene

ene

ogene

ogene

ogene

ene

ogene

ogene

ene

ogene

ogene

ogene

ene

ene

ogene

ogene

ene
