# Supplementary material for: Identifying gene expression profiles associated with neurogenesis and inflammation in the human subependymal zone from development through aging
Source: Sci Rep. 2022 Jan 7;12:40. doi: 10.1038/s41598-021-03976-4 (PMC8742079; doi:10.1038/s41598-021-03976-4)
Supplement: Supplementary file 4 — Supplementary Table 6. [file 41598_2021_3976_MOESM4_ESM.pdf]

ENSG00000005001	PRSS22	protein_coding
ENSG00000007952	NOX1	protein_coding
ENSG00000018280	SLC11A1	protein_coding
ENSG00000039537	C6	protein_coding
ENSG00000047457	CP	protein_coding
ENSG00000070915	SLC12A3	protein_coding
ENSG00000075886	TUBA3D	protein_coding
ENSG00000090104	RGS1	protein_coding
ENSG00000090339	ICAM1	protein_coding
ENSG00000095917	TPSD1	protein_coding
ENSG00000096060	FKBP5	protein_coding
ENSG00000100985	MMP9	protein_coding
ENSG00000101187	SLCO4A1	protein_coding
ENSG00000101440	ASIP	protein_coding
ENSG00000102970	CCL17	protein_coding
ENSG00000103569	AQP9	protein_coding
ENSG00000104499	GML	protein_coding
ENSG00000105281	SLC1A5	protein_coding
ENSG00000105352	CEACAM4	protein_coding
ENSG00000105697	HAMP	protein_coding
ENSG00000106366	SERPINE1	protein_coding
ENSG00000106927	AMBP	protein_coding
ENSG00000108405	P2RX1	protein_coding
ENSG00000115297	TLX2	protein_coding
ENSG00000115590	IL1R2	protein_coding
ENSG00000118113	MMP8	protein_coding
ENSG00000123843	C4BPB	protein_coding
ENSG00000124343	XG	protein_coding
ENSG00000124731	TREM1	protein_coding
ENSG00000124935	SCGB1D2	protein_coding
ENSG00000124939	SCGB2A1	protein_coding
ENSG00000125144	MT1G	protein_coding
ENSG00000125538	IL1B	protein_coding
ENSG00000127507	EMR2	protein_coding
ENSG00000127530	OR7C1	protein_coding
ENSG00000132965	ALOX5AP	protein_coding
ENSG00000134240	HMGCS2	protein_coding
ENSG00000135245	HILPDA	protein_coding
ENSG00000135373	EHF	protein_coding
ENSG00000136286	MYO1G	protein_coding
ENSG00000136315	RP11-84C10.2	lincRNA
ENSG00000137225	CAPN11	protein_coding
ENSG00000137462	TLR2	protein_coding
ENSG00000137757	CASP5	protein_coding
ENSG00000138347	MYPN	protein_coding
ENSG00000138483	CCDC54	protein_coding

ENSG00000142748	FCN3	protein_coding
ENSG00000142973	CYP4B1	protein_coding
ENSG00000143110	C1orf162	protein_coding
ENSG00000143226	FCGR2A	protein_coding
ENSG00000143546	S100A8	protein_coding
ENSG00000145642	FAM159B	protein_coding
ENSG00000147183	CPXCR1	protein_coding
ENSG00000147689	FAM83A	protein_coding
ENSG00000148926	ADM	protein_coding
ENSG00000150337	FCGR1A	protein_coding
ENSG00000151650	VENTX	protein_coding
ENSG00000155659	VSIG4	protein_coding
ENSG00000156194	PPEF2	protein_coding
ENSG00000158714	SLAMF8	protein_coding
ENSG00000160282	FTCD	protein_coding
ENSG00000160862	AZGP1	protein_coding
ENSG00000162006	MSLNL	protein_coding
ENSG00000162383	SLC1A7	protein_coding
ENSG00000163220	S100A9	protein_coding
ENSG00000163221	S100A12	protein_coding
ENSG00000163914	RHO	protein_coding
ENSG00000164362	TERT	protein_coding
ENSG00000165507	C10orf10	protein_coding
ENSG00000166492	FAM86GP	unprocessed_pseudogene
ENSG00000166523	CLEC4E	protein_coding
ENSG00000167208	SNX20	protein_coding
ENSG00000167549	CORO6	protein_coding
ENSG00000167772	ANGPTL4	protein_coding
ENSG00000168070	C11orf85	protein_coding
ENSG00000168126	OR2W6P	unprocessed_pseudogene
ENSG00000169385	RNASE2	protein_coding
ENSG00000169397	RNASE3	protein_coding
ENSG00000169507	SLC38A11	protein_coding
ENSG00000170298	LGALS9B	protein_coding
ENSG00000170458	CD14	protein_coding
ENSG00000170577	SIX2	protein_coding
ENSG00000170807	LMOD2	protein_coding
ENSG00000171051	FPR1	protein_coding
ENSG00000171236	LRG1	protein_coding
ENSG00000171346	KRT15	protein_coding
ENSG00000172538	FAM170B	protein_coding
ENSG00000173976	RAX2	protein_coding
ENSG00000174016	FAM46D	protein_coding
ENSG00000174715	RP11-79L9.2	processed_pseudogene
ENSG00000174837	EMR1	protein_coding
ENSG00000175315	CST6	protein_coding

ENSG00000176387	HSD11B2	protein_coding
ENSG00000177575	CD163	protein_coding
ENSG00000179593	ALOX15B	protein_coding
ENSG00000181227	RP4-682C21.2	processed_pseudogene
ENSG00000181433	SAGE1	protein_coding
ENSG00000182586	LINC00334	lincRNA
ENSG00000182854	OR4F15	protein_coding
ENSG00000183318	SPDYE4	protein_coding
ENSG00000184258	CDR1	protein_coding
ENSG00000184302	SIX6	protein_coding
ENSG00000185087	FAM169B	protein_coding
ENSG00000185775	SPATA31A6	protein_coding
ENSG00000186439	TRDN	protein_coding
ENSG00000187193	MT1X	protein_coding
ENSG00000187922	LCN10	protein_coding
ENSG00000188101	ALOX15P2	unprocessed_pseudogene
ENSG00000188488	SERPINA5	protein_coding
ENSG00000189143	CLDN4	protein_coding
ENSG00000189377	CXCL17	protein_coding
ENSG00000196136	SERPINA3	protein_coding
ENSG00000196183	RPS2P4	processed_pseudogene
ENSG00000196616	ADH1B	protein_coding
ENSG00000197249	SERPINA1	protein_coding
ENSG00000197251	LINC00336	lincRNA
ENSG00000197272	IL27	protein_coding
ENSG00000197658	SLC22A24	protein_coding
ENSG00000198685	LINC01565	lincRNA
ENSG00000203286	Metazoa_SRP	misc_RNA
ENSG00000203496	RP11-291L22.4	lincRNA
ENSG00000203729	LINC00272	lincRNA
ENSG00000203786	KPRP	protein_coding
ENSG00000204020	LIPN	protein_coding
ENSG00000204188	GGNBP1	unitary_pseudogene
ENSG00000204434	POTEKP	unprocessed_pseudogene
ENSG00000204460	AC079586.1	lincRNA
ENSG00000204577	LILRB3	protein_coding
ENSG00000204936	CD177	protein_coding
ENSG00000205358	MT1H	protein_coding
ENSG00000205364	MT1M	protein_coding
ENSG00000205622	AF064858.6	lincRNA
ENSG00000205628	LINC01446	lincRNA
ENSG00000206181	TCEB3B	protein_coding
ENSG00000211752	TRBV27	TR_V_gene
ENSG00000212933	KRTAP12-4	protein_coding
ENSG00000213014	VN2R17P	processed_pseudogene
ENSG00000213065	RP3-431P23.2	processed_pseudogene

ENSG00000213088	ACKR1	protein_coding
ENSG00000213302	RP11-560I19.2	processed_pseudogene
ENSG00000213549	AC005077.8	processed_pseudogene
ENSG00000213740	SERBP1P1	processed_pseudogene
ENSG00000213862	CTD-2270N23.1	processed_pseudogene
ENSG00000213997	PGAM1P7	processed_pseudogene
ENSG00000215284	RP11-198M15.1	processed_pseudogene
ENSG00000217929	CICP18	processed_pseudogene
ENSG00000218227	RP11-889L3.1	processed_pseudogene
ENSG00000218265	RPS4XP7	processed_pseudogene
ENSG00000218347	HNRNPA1P1	processed_pseudogene
ENSG00000219253	RPS6P7	processed_pseudogene
ENSG00000221102	SNORA11B	snoRNA
ENSG00000221864	KRTAP12-2	protein_coding
ENSG00000223504	RP11-542F9.1	lincRNA
ENSG00000223542	RP1-283K11.3	antisense
ENSG00000223795	RP11-473E2.2	lincRNA
ENSG00000224273	AC005077.7	unprocessed_pseudogene
ENSG00000224458	GUSBP6	transcribed_unprocessed_pseudogene
ENSG00000224594	RPL29P19	processed_pseudogene
ENSG00000224723	GUSBP10	unprocessed_pseudogene
ENSG00000224771	ATP2B2-IT2	sense_intronic
ENSG00000224776	RP11-361A21.1	processed_pseudogene
ENSG00000224863	LINC01398	lincRNA
ENSG00000224884	AC034187.2	lincRNA
ENSG00000224904	RP5-934G17.6	processed_pseudogene
ENSG00000224911	AC015936.3	antisense
ENSG00000224931	AC152010.1	unprocessed_pseudogene
ENSG00000225656	RP5-858B6.1	lincRNA
ENSG00000225726	AC007000.10	processed_pseudogene
ENSG00000225798	AC025918.2	antisense
ENSG00000226005	RP11-464C19.3	lincRNA
ENSG00000226134	RP11-120E13.1	lincRNA
ENSG00000226277	AC105393.2	lincRNA
ENSG00000226332	RP11-157P1.4	antisense
ENSG00000226349	RP11-145A3.2	sense_intronic
ENSG00000226668	RP11-526D8.7	transcribed_unprocessed_pseudogene
ENSG00000226823	SUGT1P	unprocessed_pseudogene
ENSG00000226875	ZNF877P	unprocessed_pseudogene
ENSG00000226995	LINC00658	lincRNA
ENSG00000227038	AC005077.12	transcribed_unprocessed_pseudogene
ENSG00000227061	AC079779.7	antisense
ENSG00000227225	MTND1P14	unprocessed_pseudogene
ENSG00000227632	AC018804.6	unprocessed_pseudogene
ENSG00000227695	DNMBP-AS1	antisense
ENSG00000227726	AP001271.3	sense_intronic

ENSG00000227764	RP11-354K1.1	lincRNA
ENSG00000227779	GS1-164F24.1	processed_pseudogene
ENSG00000227887	RPS26P13	processed_pseudogene
ENSG00000227947	RP11-543D5.1	lincRNA
ENSG00000227954	TARID	antisense
ENSG00000228067	RP11-61J19.3	lincRNA
ENSG00000228683	RP11-31E13.2	lincRNA
ENSG00000228863	RP11-404F10.2	antisense
ENSG00000228877	RP11-473E2.4	lincRNA
ENSG00000228963	OR7E93P	unprocessed_pseudogene
ENSG00000228983	AC025627.7	transcribed_unprocessed_pseudogene
ENSG00000229186	ADAM1A	unitary_pseudogene
ENSG00000229241	PNPT1P1	processed_pseudogene
ENSG00000229331	GK-IT1	sense_intronic
ENSG00000229389	CTD-2022H16.1	lincRNA
ENSG00000229511	GAS2L1P1	processed_pseudogene
ENSG00000229569	RP11-481G8.2	lincRNA
ENSG00000229707	SKP1P3	processed_pseudogene
ENSG00000229817	RP11-78H18.2	processed_pseudogene
ENSG00000229899	AC084290.2	processed_pseudogene
ENSG00000230053	RP11-76N22.1	processed_pseudogene
ENSG00000230267	HERC2P4	transcribed_unprocessed_pseudogene
ENSG00000230479	AP000695.6	antisense
ENSG00000230507	RPL7AP8	processed_pseudogene
ENSG00000230781	RP1-215K18.4	unitary_pseudogene
ENSG00000230793	SMARCE1P5	processed_pseudogene
ENSG00000230804	C2orf27AP3	unprocessed_pseudogene
ENSG00000230882	AC005077.14	processed_pseudogene
ENSG00000230899	MAGEA8-AS1	antisense
ENSG00000231128	RP5-1073O3.2	antisense
ENSG00000231184	FAM58DP	processed_pseudogene
ENSG00000231246	RP5-965F6.2	lincRNA
ENSG00000231324	AP000696.2	lincRNA
ENSG00000231359	AC072052.7	processed_pseudogene
ENSG00000231390	SNX18P8	unprocessed_pseudogene
ENSG00000231612	RP11-522M21.3	antisense
ENSG00000231621	AC013264.2	antisense
ENSG00000231628	RP3-355L5.4	antisense
ENSG00000231638	AC011738.4	antisense
ENSG00000231993	EP300-AS1	antisense
ENSG00000232015	HSPE1P25	processed_pseudogene
ENSG00000232022	FAAHP1	transcribed_unprocessed_pseudogene
ENSG00000232166	RP4-799P18.5	processed_pseudogene
ENSG00000232175	RP4-659I19.1	processed_pseudogene
ENSG00000232400	RAD17P1	processed_pseudogene
ENSG00000232679	RP11-400N13.3	lincRNA

ENSG00000232841	AC098592.8	processed_pseudogene
ENSG00000232936	RP11-80H5.2	antisense
ENSG00000234537	RP11-100G15.7	unprocessed_pseudogene
ENSG00000235052	RP1-150O5.3	lincRNA
ENSG00000235097	LINC00330	lincRNA
ENSG00000235204	RP11-121A14.2	antisense
ENSG00000235297	FAUP1	processed_pseudogene
ENSG00000235304	LINC01281	lincRNA
ENSG00000235335	AC016723.4	antisense
ENSG00000235371	RP4-764D2.1	processed_pseudogene
ENSG00000235535	RP11-532N4.2	antisense
ENSG00000235623	OR7E110P	unprocessed_pseudogene
ENSG00000235641	LINC00484	lincRNA
ENSG00000235663	SAPCD1-AS1	antisense
ENSG00000236116	AC064853.2	sense_intronic
ENSG00000236146	RP11-65J3.6	processed_pseudogene
ENSG00000236152	MRPS36P1	processed_pseudogene
ENSG00000236358	RP5-827C21.2	antisense
ENSG00000236496	GPS2P1	processed_pseudogene
ENSG00000236511	LINC01231	lincRNA
ENSG00000236739	CLIC4P1	processed_pseudogene
ENSG00000236975	RP5-1065P14.2	lincRNA
ENSG00000237153	RP11-132E11.2	lincRNA
ENSG00000237268	RP13-492C18.2	transcribed_unprocessed_pseudogene
ENSG00000237322	RPL7L1P10	processed_pseudogene
ENSG00000237484	AP000476.1	lincRNA
ENSG00000237580	GCSHP3	processed_pseudogene
ENSG00000237951	PPIL1P1	processed_pseudogene
ENSG00000238005	RP11-443B7.1	lincRNA
ENSG00000238039	AF011889.2	lincRNA
ENSG00000238042	RP11-815M8.1	lincRNA
ENSG00000238059	HSPE1P21	processed_pseudogene
ENSG00000238180	AC017079.4	processed_pseudogene
ENSG00000239272	RPL21P10	processed_pseudogene
ENSG00000239333	RN7SL658P	misc_RNA
ENSG00000239593	RP11-477J21.6	antisense
ENSG00000239600	AP000797.2	processed_pseudogene
ENSG00000240014	RN7SL254P	misc_RNA
ENSG00000240639	RN7SL666P	misc_RNA
ENSG00000240963	RP11-518L10.5	antisense
ENSG00000241911	TRBVB	TR_V_pseudogene
ENSG00000242012	RP11-338L18.1	lincRNA
ENSG00000242272	AK2P2	processed_pseudogene
ENSG00000242445	RPL7AP11	processed_pseudogene
ENSG00000242860	RN7SL180P	misc_RNA
ENSG00000242971	RN7SL233P	misc_RNA

ENSG00000243207	PPAN-P2RY11	protein_coding
ENSG00000243469	RPL7P51	processed_pseudogene
ENSG00000243509	TNFRSF6B	protein_coding
ENSG00000243566	UPK3B	protein_coding
ENSG00000243914	RPL5P14	processed_pseudogene
ENSG00000243988	RPS24P17	processed_pseudogene
ENSG00000244003	RN7SL143P	misc_RNA
ENSG00000244056	RN7SL417P	misc_RNA
ENSG00000244171	PBX2P1	processed_pseudogene
ENSG00000244259	AP000797.1	processed_pseudogene
ENSG00000244289	RPS3AP35	processed_pseudogene
ENSG00000244306	LINC01296	transcribed_processed_pseudogene
ENSG00000244482	LILRA6	protein_coding
ENSG00000244578	LINC01391	lincRNA
ENSG00000244671	RN7SL280P	misc_RNA
ENSG00000244752	CRYBB2	protein_coding
ENSG00000246082	NUDT16P1	unitary_pseudogene
ENSG00000247867	CTD-2530H12.1	antisense
ENSG00000248229	RP11-366M4.12	processed_pseudogene
ENSG00000248302	BNIP3P41	transcribed_processed_pseudogene
ENSG00000248514	CTC-338M12.1	antisense
ENSG00000248525	CTD-2001E22.1	sense_overlapping
ENSG00000248624	RP11-5N11.2	sense_intronic
ENSG00000248954	RP11-304F15.4	lincRNA
ENSG00000249307	LINC01088	antisense
ENSG00000249478	CTB-49A3.5	antisense
ENSG00000249513	RP11-404I7.1	lincRNA
ENSG00000249734	RP11-332J15.1	lincRNA
ENSG00000249833	CCDC37-AS1	antisense
ENSG00000249966	CTD-2194D22.1	antisense
ENSG00000250162	CSNK1A1P3	processed_pseudogene
ENSG00000250230	RP11-855O10.2	lincRNA
ENSG00000250254	PTTG2	protein_coding
ENSG00000250400	LINC00977	lincRNA
ENSG00000250712	RP11-366M4.14	processed_pseudogene
ENSG00000250929	LINC01181	antisense
ENSG00000250989	RP11-392E22.5	processed_pseudogene
ENSG00000251139	RP11-701P16.2	antisense
ENSG00000251203	RP11-14I17.1	processed_pseudogene
ENSG00000252794	RN7SKP60	misc_RNA
ENSG00000253293	HOXA10	protein_coding
ENSG00000253349	COX6B1P6	processed_pseudogene
ENSG00000253421	ZNHIT1P1	processed_pseudogene
ENSG00000253506	NACA2	protein_coding
ENSG00000254041	RP11-574O7.1	antisense
ENSG00000254872	RP13-870H17.3	lincRNA

ENSG00000255004	RP1-68D18.3	antisense
ENSG00000255192	NANOGP8	protein_coding
ENSG00000255232	RP11-438N5.4	processed_pseudogene
ENSG00000255250	CTD-2005H7.2	lincRNA
ENSG00000255293	RP11-787P24.1	processed_pseudogene
ENSG00000255334	RP11-708L7.6	sense_intronic
ENSG00000255369	RP11-740D6.3	processed_pseudogene
ENSG00000255397	AC022182.2	processed_pseudogene
ENSG00000255400	RP13-631K18.5	lincRNA
ENSG00000255440	RP11-632K5.2	sense_intronic
ENSG00000255505	RP11-485O14.1	processed_pseudogene
ENSG00000255986	MT1JP	transcribed_unprocessed_pseudogene
ENSG00000256226	RP11-582E3.2	lincRNA
ENSG00000256443	RP11-794G24.1	lincRNA
ENSG00000256953	RP11-6B19.2	lincRNA
ENSG00000256955	RP11-417L19.2	lincRNA
ENSG00000257342	RP11-571M6.7	antisense
ENSG00000257622	RP11-44N21.4	processed_transcript
ENSG00000257904	RP11-269C4.2	antisense
ENSG00000258048	RP11-530C5.1	lincRNA
ENSG00000258227	CLEC5A	protein_coding
ENSG00000258558	RP11-159L20.2	antisense
ENSG00000258646	RP11-950C14.3	antisense
ENSG00000258710	LINC01193	lincRNA
ENSG00000258909	RP11-164C12.2	lincRNA
ENSG00000258942	RP11-255G12.2	lincRNA
ENSG00000258967	HMGN1P3	processed_pseudogene
ENSG00000259023	LINC00524	lincRNA
ENSG00000259113	RP11-406H23.2	lincRNA
ENSG00000259208	RP11-621H8.1	processed_pseudogene
ENSG00000259361	LINC00927	lincRNA
ENSG00000259414	HERC2P7	unprocessed_pseudogene
ENSG00000259465	AHCYP7	processed_pseudogene
ENSG00000259493	RP11-621H8.2	processed_pseudogene
ENSG00000259592	PRELID1P4	processed_pseudogene
ENSG00000259600	RP11-925D8.3	processed_pseudogene
ENSG00000259616	RP11-507B12.2	lincRNA
ENSG00000259683	RP11-182J1.14	unprocessed_pseudogene
ENSG00000259685	CTD-2315E11.1	lincRNA
ENSG00000259843	RP11-429P3.3	antisense
ENSG00000259906	RP11-932O9.4	lincRNA
ENSG00000260062	GOLGA2P11	transcribed_unprocessed_pseudogene
ENSG00000260156	RP11-394B2.6	antisense
ENSG00000260653	RP11-114G11.5	lincRNA
ENSG00000260683	CTD-2076M15.1	lincRNA
ENSG00000260848	CTD-2009A10.1	lincRNA

ENSG00000260861	RP4-576H24.4	protein_coding
ENSG00000260868	RP11-394I13.1	lincRNA
ENSG00000260939	RP11-467J12.3	processed_pseudogene
ENSG00000261020	RP11-744K17.1	lincRNA
ENSG00000261208	RP11-452D12.1	processed_pseudogene
ENSG00000261431	RP4-616B8.4	antisense
ENSG00000261613	RP11-20I23.13	antisense
ENSG00000261744	RP11-21B21.4	antisense
ENSG00000261904	RP11-109M19.3	unprocessed_pseudogene
ENSG00000262151	RP11-876N24.2	antisense
ENSG00000262319	CTC-457L16.2	antisense
ENSG00000262560	RP11-296A16.1	protein_coding
ENSG00000262678	RP5-1050D4.4	antisense
ENSG00000263618	RP11-527H14.4	lincRNA
ENSG00000263620	RP11-599B13.6	protein_coding
ENSG00000263846	CIAPIN1P	processed_pseudogene
ENSG00000264174	RP11-212E8.1	lincRNA
ENSG00000264791	CTC-304I17.2	antisense
ENSG00000265043	RP11-728E14.3	lincRNA
ENSG00000265190	ANXA8	protein_coding
ENSG00000265881	PDLIM1P2	processed_pseudogene
ENSG00000266554	LINC01443	lincRNA
ENSG00000266893	AC005616.1	lincRNA
ENSG00000267206	LCN6	protein_coding
ENSG00000267212	CTD-2659N19.9	lincRNA
ENSG00000267318	RP11-178C3.1	protein_coding
ENSG00000267412	CTC-265F19.2	sense_intronic
ENSG00000267653	RP1-193H18.3	lincRNA
ENSG00000267690	LDLRAD4-AS1	antisense
ENSG00000267874	CTD-2527I21.9	sense_intronic
ENSG00000267924	RP11-255H23.4	lincRNA
ENSG00000268234	FKBP4P6	processed_pseudogene
ENSG00000268500	SIGLEC5	protein_coding
ENSG00000268531	RP11-32B5.8	lincRNA
ENSG00000268549	RP11-295P9.8	antisense
ENSG00000268734	CTB-61M7.2	lincRNA
ENSG00000268738	HSFX2	protein_coding
ENSG00000269825	CTD-3099C6.9	sense_intronic
ENSG00000270016	RP11-932O9.8	antisense
ENSG00000270120	RP11-327F22.6	sense_intronic
ENSG00000270136	MINOS1-NBL1	protein_coding
ENSG00000270164	LINC01480	lincRNA
ENSG00000270190	RP11-803D5.4	lincRNA
ENSG00000270614	CTC-325H20.7	processed_pseudogene
ENSG00000270775	AP000436.4	processed_pseudogene
ENSG00000271283	CTC-412M14.6	processed_pseudogene

ENSG00000271329	RP5-891H21.5	processed_pseudogene
ENSG00000271856	LINC01215	lincRNA
ENSG00000272908	RP11-121A8.1	lincRNA
ENSG00000273025	CELF6	protein_coding
ENSG00000273160	RP11-104L21.3	lincRNA
ENSG00000273259	SERPINA3	protein_coding
ENSG00000273338	RP11-386I14.4	antisense
ENSG00000273540	RP11-133L19.3	sense_intronic
ENSG00000274080	CTA-315H11.2	sense_intronic
ENSG00000274214	RP11-757O6.6	processed_pseudogene
ENSG00000274515	CTD-2026K11.5	antisense
ENSG00000275465	RP11-569G13.3	protein_coding
ENSG00000275718	CCL15	protein_coding
ENSG00000275830	RP11-403A3.3	lincRNA
ENSG00000275954	TBC1D3F	protein_coding
ENSG00000276047	Metazoa_SRP	misc_RNA
ENSG00000276269	RP11-403A3.2	lincRNA
ENSG00000276362	RP11-241M13.2	unprocessed_pseudogene
ENSG00000277708	PGBD4P2	unprocessed_pseudogene
ENSG00000277941	MIAT_exon5_1	misc_RNA
ENSG00000278001	RP11-157L3.11	processed_pseudogene
ENSG00000278212	MAFIP	unprocessed_pseudogene
ENSG00000278456	RP11-66B24.9	antisense
ENSG00000278599	TBC1D3E	protein_coding
ENSG00000278631	RP11-71B7.1	processed_pseudogene
ENSG00000278920	RP3-412A9.17	sense_overlapping
ENSG00000279365	KB-176G8.1	TEC
ENSG00000279387	CTC-525D6.3	TEC
ENSG00000279431	RP11-218M11.7	TEC
ENSG00000279530	AC092881.1	protein_coding
ENSG00000279568	RP11-20I23.5	TEC
ENSG00000279844	RP11-20F18.1	TEC
ENSG00000279878	RP11-286N22.16	TEC
ENSG00000279881	RP11-513O13.1	TEC
ENSG00000279949	RP11-10A14.9	lincRNA
ENSG00000279994	RP11-312A15.2	TEC
ENSG00000280029	CH17-140K24.2	antisense
ENSG00000280160	RP11-196G11.3	TEC
ENSG00000280260	AC017028.10	protein_coding
ENSG00000280423	RP11-299J5.1	TEC
ENSG00000281007	AL158147.2	protein_coding
